# Transfer learning enhanced water-enabled electricity generation in highly oriented graphene oxide nanochannels

**DOI:** 10.1038/s41467-022-34496-y

**Published:** 2022-11-10

**Authors:** Ce Yang, Haiyan Wang, Jiaxin Bai, Tiancheng He, Huhu Cheng, Tianlei Guang, Houze Yao, Liangti Qu

**Affiliations:** Key Laboratory of Organic Optoelectronics & Molecular Engineering, Ministry of Education, Department of Chemistry, State Key Laboratory of Tribology in Advanced Equipment (SKLT), Laboratory of Flexible Electronics Technology, Tsinghua University, 100084 Beijing, P. R. China

**Keywords:** Energy harvesting, Nanoscale materials, Electronic properties and devices, Two-dimensional materials

## Abstract

Harvesting energy from spontaneous water flow within artificial nanochannels is a promising route to meet sustainable power requirements of the fast-growing human society. However, large-scale nanochannel integration and the multi-parameter coupling restrictive influence on electric generation are still big challenges for macroscale applications. In this regard, long-range (1 to 20 cm) ordered graphene oxide assembled framework with integrated 2D nanochannels have been fabricated by a rotational freeze-casting method. The structure can promote spontaneous absorption and directional transmission of water inside the channels to generate considerable electric energy. A transfer learning strategy is implemented to address the complicated multi-parameters coupling problem under limited experimental data, which provides highly accurate performance optimization and efficiently guides the design of 2D water flow enabled generators. A generator unit can produce ~2.9 V voltage or ~16.8 μA current in a controllable manner. High electric output of ~12 V or ~83 μA is realized by connecting several devices in series or parallel. Different water enabled electricity generation systems have been developed to directly power commercial electronics like LED arrays and display screens, demonstrating the material’s potential for development of water enabled clean energy.

## Abstract (web summary)

Large-scale nanochannel integration and the multi-parameter coupling restrictive influence on electric generation are big challenges for effective energy harvesting from spontaneous water flow within artificial nanochannels. Here, authors apply transfer learning to overcome these and design optimized water-enabled generators.

## Introduction

Harvesting sustainable energy in spontaneous natural processes such as moisture diffusion^1,2^, water flow or evaporation^3–5^, and heat transfer^6,7^ has attracted extensive attention, which will potentially supply innovative and clean power for the fast-growing human society. Recently, based on the biomimetic and selective ion transport behaviors, some specific materials (e.g. MoS_2_^8^, metal-organic framework^9^, polyimide^10^, etc.) with artificial micro/nanochannels demonstrate promising electricity generation ability when water flows through them. These water flow-enabled electric generators (WEGs) are proposed to involve the mechanism of the electric double layer (EDL) at the solid–liquid interface and water flow-induced charge separation^11–13^. Meanwhile, the energy conversion ability has been improved by regulating the intrinsic or structural feature (e.g. surface charge density, channel size)^14,15^ of materials. However, the development of high-performance WEG still faces great challenges and it seems to encounter an insurmountable bottleneck in macro-scale practical applications. The main reasons are as follows: (1) Although nanoscale WEGs have favorable power density, it is difficult to maintain the energy generation ability after integrating nanochannels into macroscopic assemblies, because of the disordered structures and incoordination between individual nanochannels^16–18^. (2) In addition to the macroscale and uniform integration of nanochannels, the performance of WEG will also strongly depend on the regulation of surface charges of nanochannels, interactions between water and materials, internal water transport and so on^3,13,19,20^. Unfortunately, the multiple factors are restrictively coupled, and the change in one of them will dramatically affect the matching configuration and ultimate power generation ability^21,22^. Especially in the expected macroscopic WEG, the interrelationship among massive nanochannels will bring multi-objective trade-offs, multi-parameter coupling, and vast parameter optimization exploration, which could make the experiments complicated, expensive, and time-consuming^14,23,24^. (3) To promote water flow through the nanochannels of WEGs, a large water pressure difference should be applied on two sides of the nanochannels, which makes the energy conversion system complex and is far from the expected natural energy transfer process^5,11,15,25,26^. Besides, the mechanical properties of WEG should meet the practical requirements in various conditions^27–29^. All these problems have severely restricted the fabrication and development of macroscopic WEG to achieve the realistically available applications.

2D graphene oxide (GO) sheets rich in oxygenated functional groups can be easily layer-by-layer assembled into desirable and oriented structures with the forming capability in macroscale^30,31^. Meanwhile, the versatile modification of functional groups on GO sheets can control the surface charge density when interacting with water^32,33^. As a result, abundant and designable 2D nanochannels between GO sheets could be uniformly integrated into macroscopic assemblies, which provide ideal platforms for the development of high-performance WEG^34,35^.

**Fig. 1:**
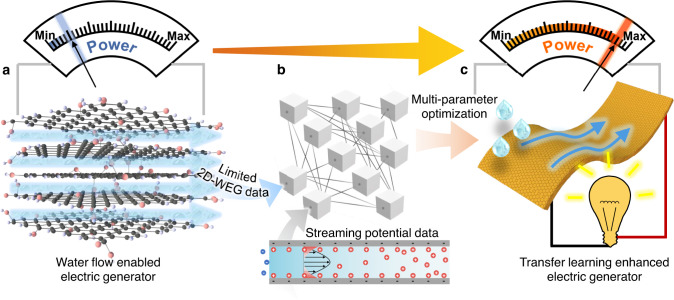
TL enhanced 2D-WEG with integrated 2D channels. **a** A 2D-WEG based on the GO assembled framework with massive integrated 2D channels can generate considerable electric energy by the spontaneous internal water flow. **b** To solve the multi-parameters coupling optimization problem of the developed 2D-WEG, the TL framework is implemented to achieve accurate prediction and optimization of generation performance using limited experimental data and abundant streaming potential data with similar backgrounds. **c** Ultimately, the TL-optimized 2D-WEGs with mechanical flexibility and enhanced power generation performance is produced to power a variety of commercial appliances.

In this work, WEG with massive integrated 2D nanochannels (2D-WEG) within the long-range (1–20 cm) oriented 2D GO assembled framework is developed by a rotational freeze-casting method. This 2D-WEG with tunable inner structural and chemical characters can spontaneously absorb water and promote water flow inside nanochannels to generate considerable electric energy (Fig. 1a). Especially, we implement a transfer learning (TL) strategy to address the complicated multi-parameters coupling optimization for the 2D-WEG with limited experimental data (Fig. 1b)^36,37^. Unlike previously reported single-parameter analyses, this TL strategy can provide uniform multi-parameter modeling and highly accurate performance prediction under a small experimental dataset, which is a high-efficiency route for the design of 2D-WEG. In consequence, the TL-optimized 2D-WEG generates ~2.9 V voltage or ~16.8 μA current (Fig. 1c). In addition, the 2D-WEG with favorable mechanical stability can be flexibly designed as a waterscape screen as well as the water power generation folding fan and building component to generate the considerable electricity. Moreover, the high electric output of ~12 V or ~83 μA is realized by connecting several 2D-WEGs in series or parallel to directly power commercial electronics like calculators, LED arrays, and display screens, demonstrating the potential of TL-empowered 2D-WEG for the development of water enabled clean energy system.

## Results

### Rotational freeze-casting method for the preparation of 2D-WEG

**Fig. 2:**
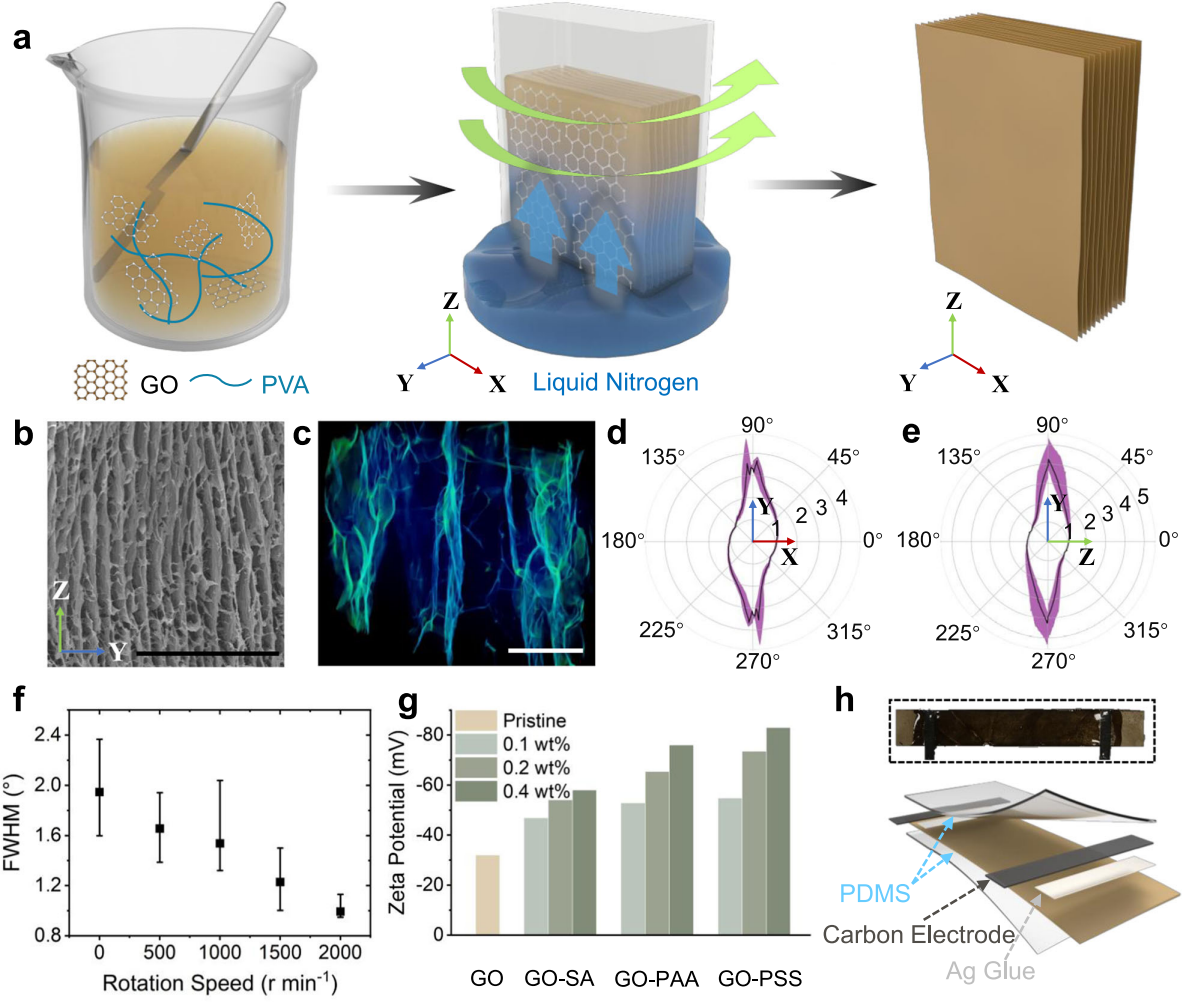
Preparation and characterization of 2D-WEG. **a** Schematic illustration of the preparation process of GO assembled framework by the rotational freeze-casting method. **b** The cross-section scanning electron microscope (SEM) image of GO assembled framework taken along the **X**-axis directions. Scale bar: 500 μm. **c** The 3D structure reconstructed from X-ray tomography images of the GO assembled framework, which shows the GO sheets (indicated by the bright blue area) arranged in space (black area) parallel to the **X**–**Z** plane. Scale bar: 50 μm. The value of tortuosity in different directions in **d** **X**–**Y** plane and **e** **Y**–**Z** plane, where the purple area represents the interval between the maximum and minimum tortuosity in different cross-sections of the entire GO assembled framework, and the black line indicates the average tortuosity over all cross-sections. **f** The FWHM of XRD patterns of the GO assembled framework prepared by using different rotational speeds and the mean value and error bar are calculated from multiple samples. **g** Zeta potential of GO assembled framework with different polyelectrolyte molecules contents. **h** Schematic of the structure of a 2D-WEG. Inset h is an optical photograph of a 2D-WEG. Source data are provided as a Source Data file.

The 2D-WEG with long-range and integrated 2D nanochannels is prepared by using the rotational freeze-casting method (Fig. 2a). In this process, high-speed rotation treatment generates strong centrifugal force in the radial direction, which will cause GO sheets to align in the tangential direction of mold (**X**-axis direction)^38^. Meanwhile, the expelling force of the directional ice crystals from the bottom makes GO sheets arrangement along with the vertical direction (**Z**-axis direction)^39^. As a result, GO sheets can be assembled orderly and the long-range texture parallel to **X**–**Z** 2D plane is formed along the long side of the employed mold (Supplementary Fig. 1). In contrast to previous porous graphene assemblies that have random structures or ordered distribution in only one dimension^31,39^, this GO assembled framework can arrange GO sheets parallel to the **X**–**Z** plane, which forms the massive oriented 2D channels (Fig. 2b and Supplementary Fig. 2). X-ray tomography images and the 3D reconstruction results clearly show the 2D lamellar structure of GO sheets (Fig. 2c). Furthermore, the tortuosity value in the **Y** direction is ~4.5 that is much higher than the value (~1) in **X** and **Z** directions, further indicating the ordered arrangement of GO sheets in **X**–**Z** plane (Fig. 2d and e)^40^. Meanwhile, the orientation degree of GO sheets can be regulated by controlling the rotational speed in the preparation of a 2D GO assembled framework (Supplementary Fig. 3), which is reflected in the X-ray diffraction (XRD) results. The full width at the half maximum (FWHM) of XRD patterns decreases with increasing rotational speed, indicating that the distribution of GO sheets is well-ordered (Fig. 2f)^38^. Additionally, the surface charge density of GO sheets will affect the water-enabled power generation process, which can be controlled with the modification of different polyelectrolyte molecules^3,33^. The introduction of polystyrene sulfonate (PSS), polyacrylic acid (PAA), and sodium alginate (SA) with sulfonic/carboxyl/hydroxyl groups can modulate the surface charge density of GO sheets as indicated by the decrease in Zeta potential from −31 to −83 mV (Fig. 2g), which provide the wide optimizable space for the material and device (Supplementary Figs. 4 and 5). Finally, the 2D GO assembled framework is tableted along the **Y**-axis direction and processed by the direct laser writing into the designed shape for the 2D-WEG packaging. Then, the electric conductive carbon electrodes are subsequently connected at the ends of the 2D GO assembled framework to collect the electric signal, and PDMS is coated on the outside to confine the physical space of the GO assembled framework and maintain the mechanical strength (Fig. 2h and Supplementary Figs. 6, 7). The mechanically stable and macroscopic 2D-WEG integrates massive 2D-oriented nanochannels, while the key parameters related to power generation can be easily adjusted in the preparation process.

### Water-enabled electric generation and transfer learning optimization

**Fig. 3:**
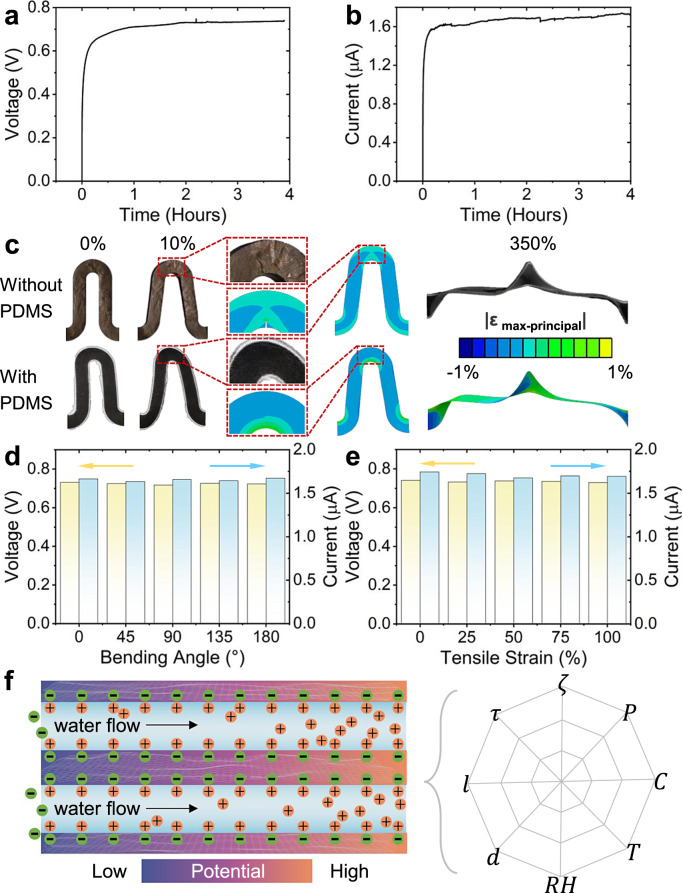
Electric output performance of 2D-WEG. **a** *V*_oc_ and **b** *I*_sc_ of an as-prepared 2D-WEG. **c** Optical images and finite-element analysis results of serpentine-structured GO assembled frameworks with and without PDMS coating, which was stretched by 0% (initial state), 10%, and 350%. The locally enlarged views of finite-element analysis results and optical photos show that the GO-assembled framework without PDMS coating has obvious damage due to stress concentration under 10% tensile deformation, while the GO-assembled framework with PDMS coating is not damaged by stress concentration. Meanwhile, the maximum strain is still less than the critical strain at a break under 350% tensile deformation. *V*_oc_ output and *I*_sc_ output under different **d** bending and **e** stretching conditions. **f** Schematic illustration of the energy conversion process, where the directional transport of water leads to an electrical potential difference between the two ends of the 2D-WEG and the right figure indicates the eight characteristic parameters that affect the energy conversion process, including Zeta potential $\zeta$, structure tortuosity $\tau$, device length *l*, nanochannel spacing *d*, environmental relative humidity *RH*, liquid temperature *T*, ion’s concentration *C* and driving pressure *P*. Source data are provided as a Source Data file.

Because of the abundant oxygenated functional groups and uniformly massive 2D nanochannels, when water is supplied at one edge of 2D-WEG, water can be spontaneously absorbed and flowed inside the massive 2D nanochannels under the action of capillary force to generate electricity (Supplementary Fig. 8). The as-prepared 2D-WEG can produce an open‐circuit voltage (*V*_oc_) of ~0.73 V (Fig. 3a) and a short‐circuit current (*I*_sc_) of ~1.68 μA (Fig. 3b). The *V*_oc_ and *I*_sc_ of the 2D-WEG spontaneously last for hours without significant attenuation (Supplementary Fig. 9), because water can diffuse along the 2D nanochannels to the other edge of 2D-WEG and evaporate continuously to the air. Meanwhile, the rational PDMS package makes the 2D-WEG self-supporting and bendable, effectively avoiding damage due to stress concentration (Fig. 3c and Supplementary Fig. 10)^41^. Under different bending deformations, the *V*_oc_ and *I*_sc_ fluctuations of the 2D-WEG are within ±0.03 V and ±0.05 μA, respectively (Fig. 3d). The 2D-WEG with a serpentine form maintains structural stability at 100% tensile deformation and water can transmit along the designed path to produce electricity under the initial or stretching state. Also, the generated *V*_oc_ and *I*_sc_ only fluctuate by approximately ±0.04 V and ±0.07 μA, respectively, which shows stable power generation and mechanical flexibility in varied deformation environments (Fig. 3e).

The mechanism for water-enabled electricity generation of 2D-WEG is proposed as follows. First, the oxygenated functional groups (e.g. carboxy) on the GO sheets will be ionized after adsorbing water, which makes 2D GO nanochannels negatively charged^42^. Meanwhile, the positively charged ions in the water will adsorb on the surface of GO sheets to form EDL^3,24,27,42,43^. Spontaneous water absorption at one edge and evaporation at the other edge will result in directional water flow inside the 2D-WEG. Then, with the directional transport of water in the 2D-WEG, the positive ions adsorbed in the EDL will move towards the evaporation side due to the shearing effect of water flow, while the negative ions can hardly pass through positively charged nanochannels, thus forming the electric potential difference between the two electrodes (Fig. 3f)^11,12,44–46^. Briefly, the electricity generation mechanism of 2D-WEG should be concluded as the charge separation induced electric potential difference caused by the directional water flow in 2D nanochannels (Supplementary Figs. 11 and 12)^3,42^. Therefore, the mechanism of 2D-WEG is mainly based on the reported streaming potential^5,11^, which is the phenomenon of the liquid flowing through the charged nanochannels with EDL and inducing positive and negative charge separation, then forming the electric potential difference. However, the electricity generation of 2D-WEG involves the restrictive coupling of several processes such as water absorption, directional flow, and evaporation, which is beyond the only streaming potential process of water transport^20,46^. More parameters that affect the performance of 2D-WEG should be considered systematically. For example, liquid temperature (*T*) and environmental relative humidity (RH) would affect the water evaporation process; Ion concentration (*C*) in water and Zeta potential ($\zeta$) of the 2D-WEG would affect the EDL; The device length (*l*), the nanochannel spacing (*d*), structure tortuosity ($\tau$) of 2D-WEG and driving pressure (*P*) would affect the directional transport behavior of water^13,14,20,23,46–48^. Predictably, the performance of 2D-WEG is significantly influenced by the synergy of these parameters, and they will be restrictively coupled and involve huge trade-offs, making the experimental exploration of optimal parameter combinations challenging. Recently, machine learning techniques have demonstrated an excellent ability to model complex systems^36,37,49,50^, which depends on the training data volume. However, experimentally collecting large-scale data is complicated and time-consuming. TL is a machine learning method that utilizes knowledge implied in the related domain to improve the performance of machine learning models with insufficient training data. Based on the highly similar basic background (e.g., EDL formation and charges separation) behind 2D-WEG and streaming potential generation, we transfer the deep knowledge representation from the abundant streaming potential data to efficiently guide the design of 2D-WEG with limited experimental data^36,37^. This TL strategy could greatly reduce the amount of experimental data required by reusing data with similar backgrounds, thus significantly lowering the threshold for applying machine learning methods to optimize the performance in complex 2D-WEG systems.

**Fig. 4:**
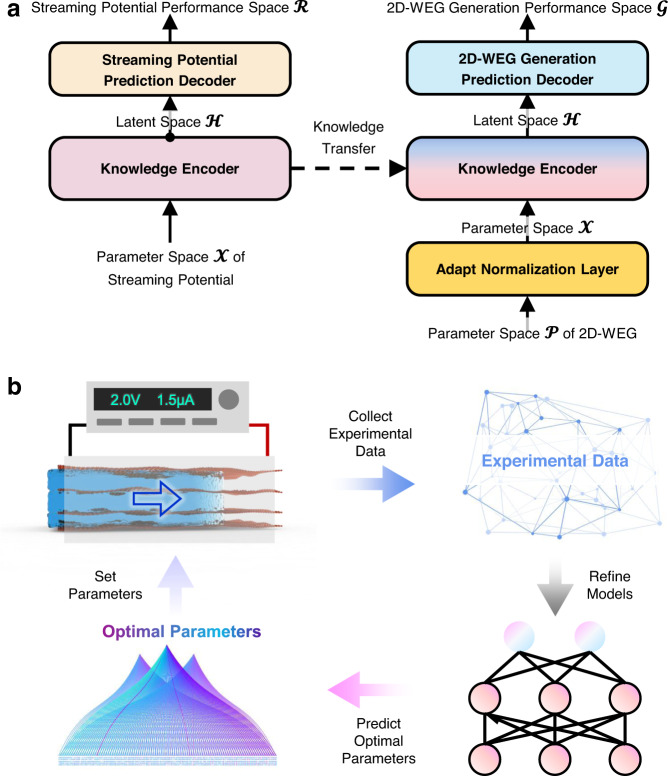
TL framework and iterative optimization strategy. **a** Schematic illustration of the TL framework. A source model with an encoder-decoder architecture was first trained using streaming potential data. Then, the Encoder is transferred to the opt-model to initialize the parameters, and the experimental data are used to train decoding predictions from the latent space to 2D-WEGs performance. **b** Schematic illustration of the iterative optimization process. Initially, the generation performance data of 2D-WEGs with different structural parameters are collected experimentally. The newly collected data and the previous data are then used to refine the opt-models. Subsequently, a differential evolution algorithm is used to obtain several new combinations of structural parameters based on the predictions of opt-models, and reconstruct 2D-WEGs, and collect experimental data accordingly.

To apply the TL strategy, we first learn knowledge representation in the streaming potential domain (Fig. 4a). The source model$:{{{{{{{\boldsymbol{{{{{\mathcal{X}}}}}}}}}}}}}\to {{{{{{{{\boldsymbol{{{{{\mathcal{R}}}}}}}}}}}}}}$ is a mapping from the parameter space ${{{{{{{{\boldsymbol{{{{{\mathcal{X}}}}}}}}}}}}}}$ of streaming potential to the generation performance space ${{{{{{{{\boldsymbol{{{{{\mathcal{R}}}}}}}}}}}}}}$, which consists of two components—Encoder and Decoder. The Encoder$:{{{{{{{{\boldsymbol{{{{{\mathcal{X}}}}}}}}}}}}}}\to {{{{{{{{\boldsymbol{{{{{\mathcal{H}}}}}}}}}}}}}}$ embeds the raw parameters $\vec{{{{{{{{\bf{x}}}}}}}}}\in {{{{{{{{\boldsymbol{{{{{\mathcal{X}}}}}}}}}}}}}}$ of the streaming potential into the latent space ${{{{{{{{\boldsymbol{{{{{\mathcal{H}}}}}}}}}}}}}}$ by using a 4-layer multilayer perceptron (MLP). The Decoder$:{{{{{{{{\boldsymbol{{{{{\mathcal{H}}}}}}}}}}}}}}\to {{{{{{{{\boldsymbol{{{{{\mathcal{R}}}}}}}}}}}}}}$ uses the knowledge representation $\vec{{{{{{{{\bf{h}}}}}}}}}$ in the latent space ${{{{{{{{\boldsymbol{{{{{\mathcal{H}}}}}}}}}}}}}}$ to predict the streaming potential performance $\vec{{{{{{{{\bf{r}}}}}}}}}\in {{{{{{{{\boldsymbol{{{{{\mathcal{R}}}}}}}}}}}}}}$ with a 2-layer MLP. The source model learns the relationship between the parameters of the streaming potential and the induced voltages and currents from the open dataset until convergence^51^ (Supplementary Fig. 13a). The 2D-WEG performance optimization model (opt-model)$:{{{{{{{{\boldsymbol{{{{{\mathcal{P}}}}}}}}}}}}}}\to {{{{{{{{\boldsymbol{{{{{\mathcal{G}}}}}}}}}}}}}}$ has three components——NormLayer, Encoder, Decoder. The NormLayer$:{{{{{{{{\boldsymbol{{{{{\mathcal{P}}}}}}}}}}}}}}\to {{{{{{{{\boldsymbol{{{{{\mathcal{X}}}}}}}}}}}}}}$ is a learnable adaptive normalization layer that can perform learnable element-wise affine normalization to accommodate the order of magnitude mismatch between experimental data and streaming potential data. Due to the similarity of the mechanism background, the Encoder$:{{{{{{{{\boldsymbol{{{{{\mathcal{X}}}}}}}}}}}}}}\to {{{{{{{{\boldsymbol{{{{{\mathcal{H}}}}}}}}}}}}}}$ of the opt-model is initialized using the weights of the Encoder of the source model, which is only fine-tuned during the training process. Finally, the Decoder$:{{{{{{{{\boldsymbol{{{{{\mathcal{H}}}}}}}}}}}}}}\to {{{{{{{{\boldsymbol{{{{{\mathcal{G}}}}}}}}}}}}}}$ with 3-layer task-specific MLP is deployed at the top to accurately characterize the relationship between the hidden knowledge representation $\vec{{{{{{{{\bf{h}}}}}}}}}\in {{{{{{{{\boldsymbol{{{{{\mathcal{H}}}}}}}}}}}}}}$ and the generated *V*_oc_/*I*_sc_ $\in {{{{{{{{\boldsymbol{{{{{\mathcal{G}}}}}}}}}}}}}}$. Above all, this opt-model for 2D-WEG based on TL strategy reuses the knowledge representation learned from the streaming potential data through weight sharing (see Methods section for full details about the TL framework). Furthermore, in terms of the opt-model training, an iterative optimization strategy is adopted to optimize the parameters of 2D-WEG by using the latest experimental data (Fig. 4b)^52^. The initial stage of the iterative optimization strategy is to experimentally collect 2D-WEG data to train opt-models. Then, the learned opt models are used to generate a series of candidate parameters to maximize the *V*_oc_ or *I*_sc_. Subsequently, we design the 2D-WEG based on the candidate parameters and re-collect experimental data to refine the opt-model to locally alleviate the inevitable over-fitting problem (see the Methods section for details about the iterative optimization strategy). These steps are repeated until there is no further improvement in the electricity generation performance, which takes about 30 cycles in 2D-WEG.

**Fig. 5:**
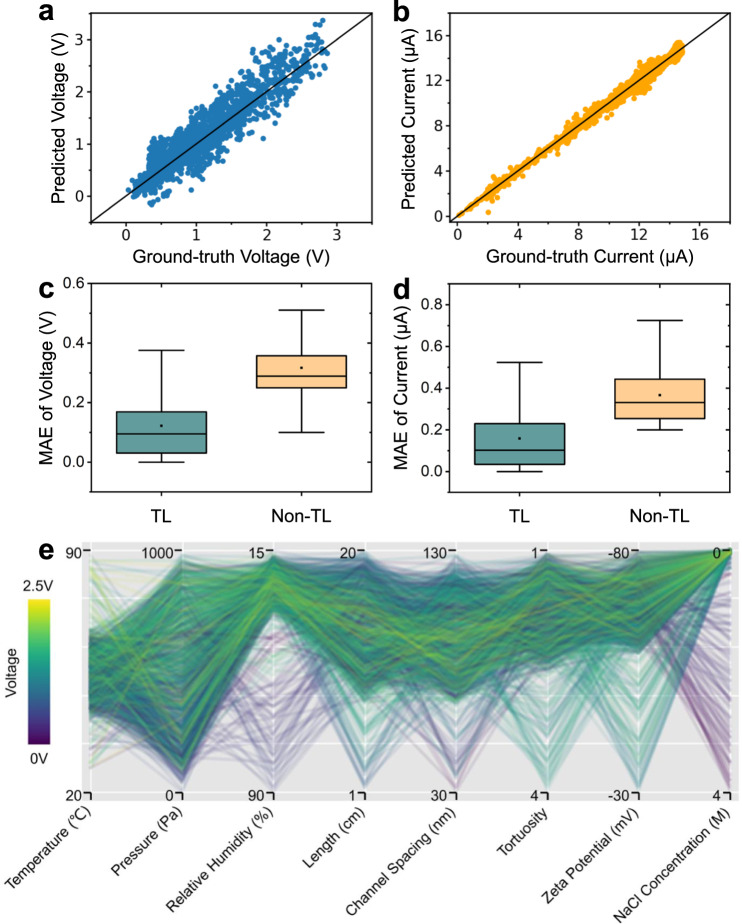
The opt-model performance for *V*_*oc*_ and *I*_*sc*_ predictions. The opt-model predicted **a** *V*_oc_ and **b** *I*_sc_ versus experimental data (ground truth) of the 2D-WEGs. Each point represents a 2D-WEG instance, where the abscissa value is the *V*_oc_/*I*_sc_ measured experimentally, and the ordinate value is the *V*_oc_/*I*_sc_ predicted by the TL-based opt-model. All points are clustered around the diagonal, indicating that the opt-model is able to make accurate predictions for 2D-WEGs with different parameters and generation performance. Box plots of the absolute errors of the predictions for **c** *V*_oc_ and **d** *I*_sc_ by the TL-based opt-model versus the absolute errors of the predictions by the model trained from scratch, where the boxes show the 25th, 50th, and 75th percentiles of absolute errors, the black dots indicate MAE, and the upper and lower bounds represent 1.5 times the interquartile range. **e** The parallel coordinates plot shows the *V*_oc_ of the 2D-WEGs with different parameter configurations. The intercept of polylines at each vertical ordinate axis indicates the value of a characteristic parameter, and the color of the polyline represents the *V*_oc_ value. Source data are provided as a Source Data file.

As shown in Fig. 5a, when only limited experimental data are used to train the TL-based opt-model, the mean absolute error (MAE) of *V*_oc_ prediction is about 0.12 V with a corresponding mean relative error of 15.0%^53^, and the correlation coefficient is 0.93. Similarly, the MAE of *I*_sc_ prediction is about 0.16 μA with a corresponding mean relative error of 5.9%, and the correlation coefficient is 0.96 (Fig. 5b), indicating that the TL-based opt-model can accurately predict the generation performance from the characteristic parameters of the 2D-WEG using limited experimental data. For the model trained from scratch without TL, the MAEs of *V*_oc_ and *I*_sc_ on the test set are expanded by 2.56 times and 2.32 times (Fig. 5c and d), respectively, which further shows that the TL-based opt-model can significantly avoid overfitting problem on small-scale training sets (Supplementary Fig. 13b and c). More importantly, unlike the previous single-factor optimization adopted for water-enabled power generation systems, this TL-based optimization strategy can systematically model and search for the best combination of multiple parameters^52^. As indicated in the parallel coordinate plot outputted by the TL-based opt-model, the corresponding relationship between the power generation performance of 2D-WEG and all the parameters (*T,* RH*, C*, $\zeta$*, l, d*, $\tau$*, P*) can be provided directly (Fig. 5e), which is crucial for exploring optimal parameter combinations in multi-parameters coupled scenarios. As a result, the TL-based opt-model can use data with a similar background to reduce the amount of training experimental data required by machine learning methods, and accurately learn the dependence of power generation performance on 2D-WEG characteristic parameters. Meanwhile, the learned opt-model is able to deliver a clear and high-precision numerical model of the relationship between the influencing factors and *V*_oc_ or *I*_sc_ of 2D-WEG to guide 2D-WEG design and help to understand how key factors influence the energy generation process (Supplementary Note 1, Table 1 and Figs. 14–17).

### Optimized 2D-WEGs for scalable integration and applications

**Fig. 6:**
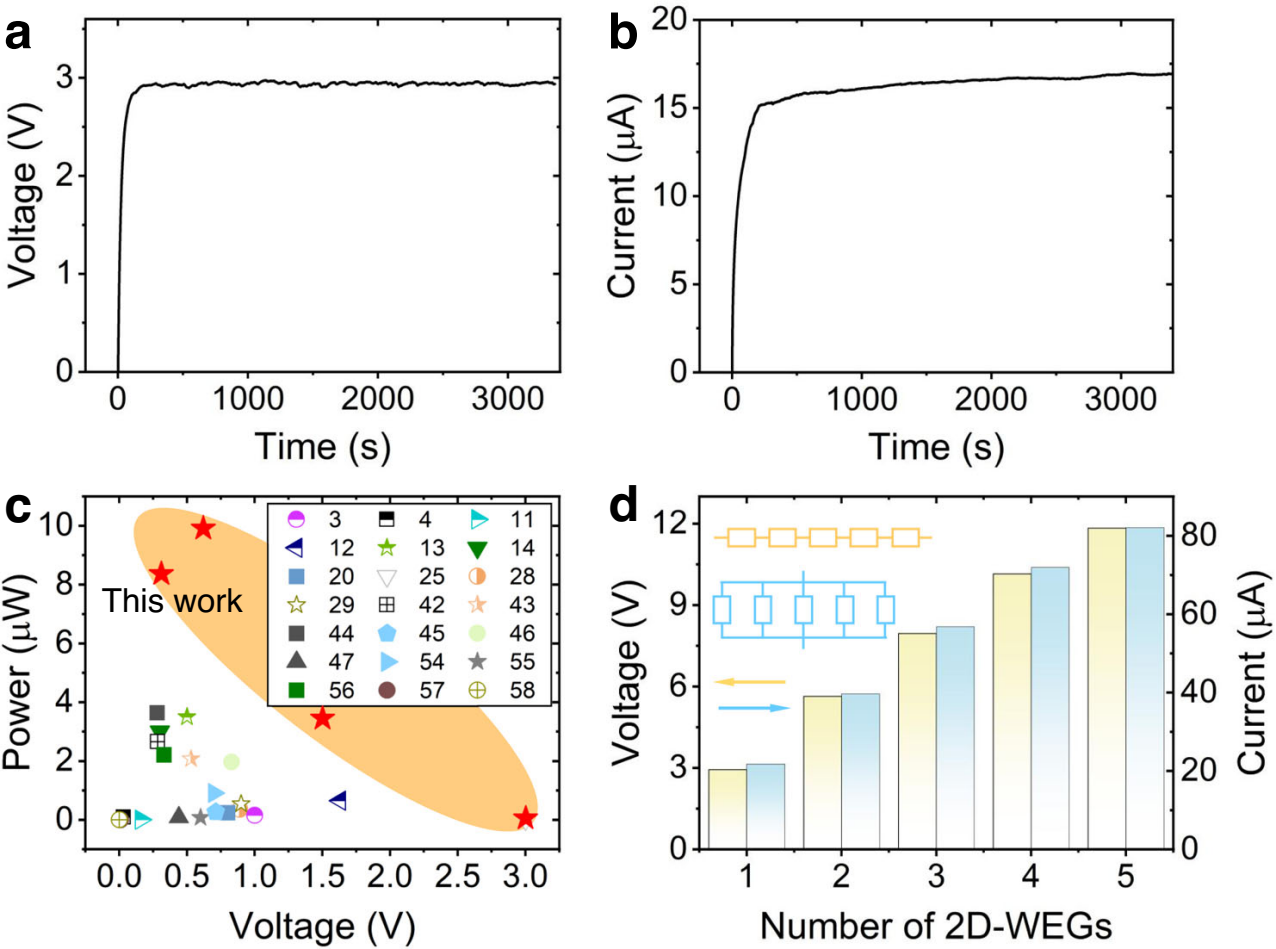
Electric output performance of optimized 2D-WEG. **a** The *V*_oc_ output produced by a single 14.6 cm length 2D-WEG designed by the opt-model. **b** The *I*_sc_ output produced by a single 1 cm length 2D-WEG. **c** Systematic performance comparison of *V*_oc_ and power output of various reported spontaneous water flow-induced power generation devices^3,4,11–14,20,25,28,29,42–47,54–58^, where the output power is calculated by multiplying the output voltage and output current. **d** The *V*_oc_ output from five 2D-WEGs connected in series and the *I*_sc_ output from five 2D-WEGs connected in parallel. Source data are provided as a Source Data file.

Benefiting from accurate modeling and multi-parameters coupling optimization of TL-based opt-model, a series of 2D-WEGs with desirable voltage and current outputs according to requirements can be prepared in a controllable manner (Supplementary Fig. 18). As shown in Fig. 6a, we design and fabricate a 14.6 cm long 2D-WEG with matched parameters (*d* = 98 nm, ${\tau }_{Y}$ = 1.04, $\zeta$ = −83 mV, *T* = 30 °C, *RH* = 15%, *P* = 0 Pa, *C* = $0{{{{{{{\rm{M}}}}}}}}$), which performs a *V*_*oc*_ of ~2.9 V that is about 397% higher than that of initially 2D-WEG. Meanwhile, a high *I*_sc_ of ~16.8 μA can be achieved by a 1 cm long 2D-WEG (Fig. 6b). Figure 6c further shows that the 2D-WEGs with featured parameters from the TL-based opt-model have higher power output at different *V*_oc_ compared to previous water flow-induced electricity-generating devices (Supplementary Table 2)^3,4,11–14,20,25,28,29,42–47,54–58^. Additionally, the power output can be further scaled up by simple series and parallel connections of 2D-WEGs. An integrated device with five 2D-WEG units connected in series can produce a voltage of ~11.9 V and a high current output of ~82.7 μA can be realized by connection in parallel (Fig. 6d). All these results confirm the integration of 2D nanochannels in macroscopic 2D-WEG to generate favorable electric power. Moreover, the developed TL can guide the performance optimization of 2D-WEG by accurate modeling and combinatorial multi-parameter output, which provides an efficient strategy for the development of macroscale water-powered systems.

**Fig. 7:**
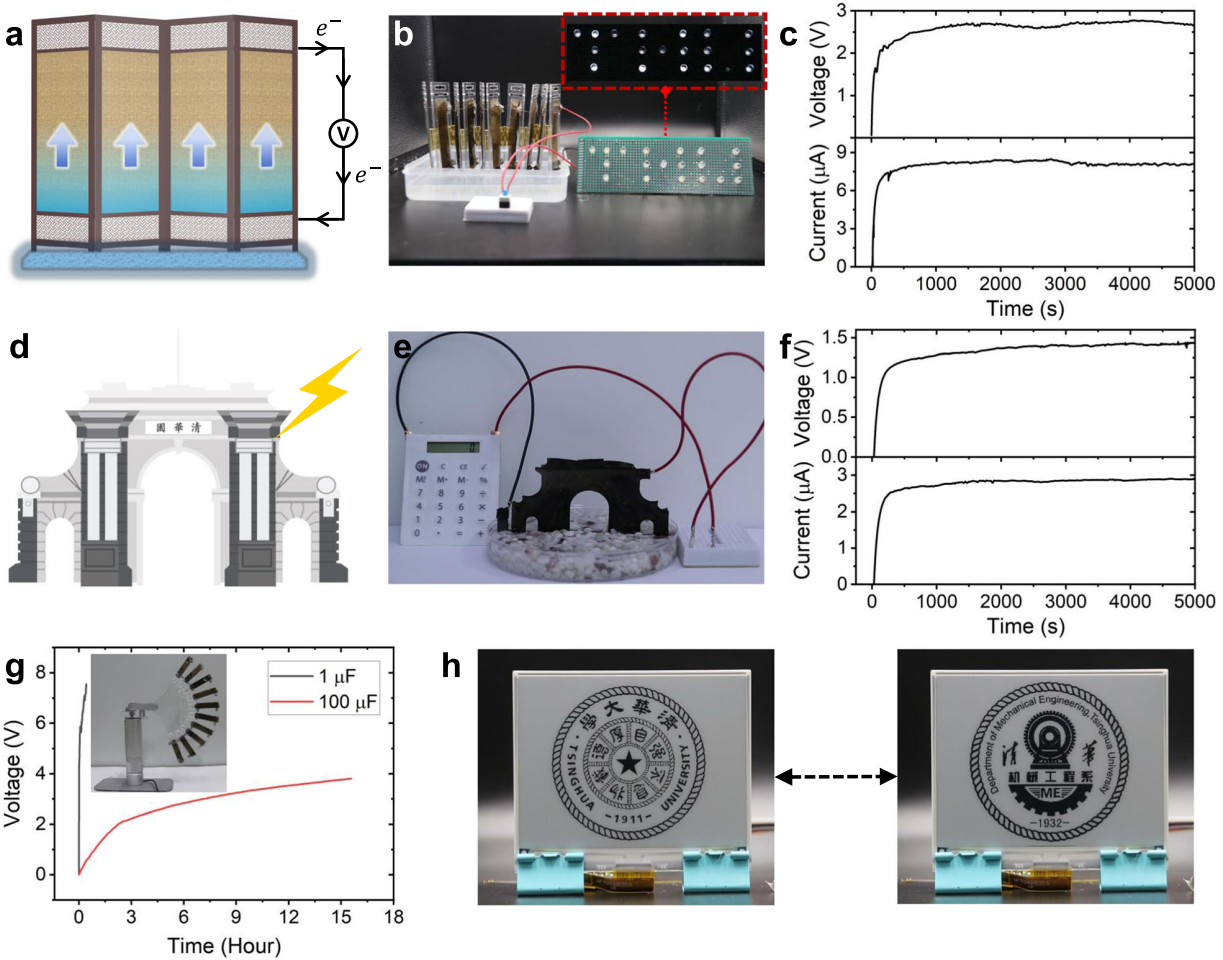
Application demonstrations of 2D-WEGs. **a** Schematic of a waterscape screen generating electricity by spontaneous water transport. **b** Optical photograph of nineteen LEDs powered by a waterscape screen with 10 integrated 2D-WEGs and the inset **b** shows 19 LEDs being lit up. **c** The *V*_oc_ and *I*_sc_ are generated by the waterscape screen with 10 integrated 2D-WEGs when the bottom is in contact with water. **d** Schematic illustration of electrical output produced by watering a small architectural landscape designed by a 2D-WEG. **e** A commercial calculator is driven by a small architectural landscape made from a single 2D-WEG. **f** The *V*_oc_ and *I*_sc_ generated by watering the architectural landscape. **g** Voltage–time curves of commercial capacitors with varying capacitance charged by waving a fan made up of ten 2D-WEGs. Inset **g** is an optical photograph of the mechanical device that automatically waves the fan with integrated 2D-WEGs to collect electrical energy. **h** The electric energy stored in the capacitor power the electronic ink screen to play several animations. Source data are provided as a Source Data file.

To demonstrate practical applications, a waterscape screen containing 10 2D-WEGs has been explored (Fig. 7a), which can spontaneously absorb water from the bottom like natural plants and continuously generate electricity (~2.8 V and ~8.3 μA) to power 19 LEDs (Fig. 7b, c and Supplementary Movie 1). Benefitting from the designability of 2D-WEG based on the TL-based opt-model, a small architectural landscape constructed from a single 2D-WEG has been further developed (Fig. 7d). As shown in Fig. 7e and f, the electricity generated by this architectural landscape can support a series of scientific calculations on a commercial calculator by simply watering it (Supplementary Movie 2). In addition, according to the positive influencing factors suggested by opt-model, the kinetic energy of the fan is further used to promote power generation by integrating 2D-WEGs on the fan blade (Fig. 7g). The water at one side of 2D-WEG can be transported towards the other side in an accelerated manner (Supplementary Fig. 19). Then, the generated power stored in commercial energy storage equipment (Fig. 7g) is enough to drive a 4.2-inch electronic ink screen to play multiple animations (Fig. 7h and Supplementary Movie 3).

## Discussion

In summary, the macroscopic 2D-WEG integrated with massive 2D-oriented nanochannels is developed to generate considerable electricity by internal spontaneous water flow. A TL strategy is implemented to efficiently guide 2D-WEG design and achieve multi-parameters coupling optimization and accurate performance prediction using limited experimental data. As a result, the 2D-WEG with mechanical flexibility can generate a high voltage of ~2.9 V or a current of ~16.8 μA, and adjust the output performance in a controllable manner according to practical requirements. The simple series or parallel connection of 2D-WEGs is able to enlarge the electricity generation up to ~11.9 V and ~ 82.7 μA, respectively. Furthermore, diverse water-enabled electricity-generating systems have been developed, including the waterscape screen, the architectural landscape, and the 2D-WEG fan, which can power scientific calculator, LED array, and electronic ink screen. The fabricated 2D-WEG and TL optimization strategy have demonstrated the potential to develop the promising water-enabled clean energy system.

## Methods

### Preparation of the 2D-WEG

GO dispersion was synthesized by using the modified Hummers’ method. To prepare the 2D-WEG with long-range and integrated 2D nanochannels, 100 mL GO dispersion (5.5 mg mL^−1^) was first mixed with 3 mL PVA solution (1 wt%) and 5 mL ethanol and the appropriate amount of polyelectrolyte molecules. The mixed dispersion was transferred to a homemade cuboid mold. After the mold rotated (2000 r min^−1^) around the **Z**-axis for 10 min, the bottom was slightly exposed to liquid nitrogen of −196 °C under the rotation. After the GO dispersion was completely frozen, the GO-assembled framework was obtained by the conventional freeze-drying method. Subsequently, a tableting process was applied to get a controllable thickness along the **Y**-axis and the direct laser writing cut the GO-assembled framework into the desired shape for device fabrication. Then, carbon paper electrodes were attached to the top and bottom of the GO-assembled framework using conductive silver glue. Finally, the device was carefully sealed with a 2-h pre-formed PDMS film to complete the 2D-WEG preparation. The PDMS was first pre-formed at 80 °C for 2 h and tightly sealed the GO assembled framework at 80 °C for 4 h to cure.

Polystyrene sulfonic acid (PSS, M.W. 75,000) was purchased from Energy Chemical Co., Ltd. (Anhui, China). Polyacrylic acid (PAA, M.W. 450,000) was from Aikon International Co., Ltd. (Jiangsu, China). Sodium alginate (SA) was purchased from Shanghai Macklin Biochemical Co., Ltd (Shanghai, China). Polyelectrolyte solution was slowly added to GO dispersion under vigorous stirring and the mixture was subjected to sonication for 30 min to obtain homogeneous dispersions. The relative content of the composites can be easily adjusted by changing the volume of the polyelectrolyte solution.

#### Electrical measurements

The electrical output signal of 2D-WEGs was measured by using a Keithley 2612 multifunctional source meter. During the test, one end of 2D-WEG is in contact with water while the other end is kept in a constant temperature and humidity chamber to ensure a stable condition. The electrochemical Impedance Spectroscopy analyses were performed on a CHI 660E electrochemical workstation (CH Instruments Inc.).

#### Characterization

The morphology of 2D-WEGs was investigated with a scanning electron microscope (SEM, FLexSEM 1000). Optical photos and videos are taken with the camera (SONY Alpha ILCE-7RM3). X-ray diffraction (XRD) patterns were recorded on a Bruker AXS D2 PHASER diffractometer with a Cu Kα irradiation source (*λ* = 1.54 Å). The 3D microstructure of 2D-WEG was examined by the X-ray nano-tomography system (ZEISS Xradia 520 Versa) and the structural tortuosity was calculated from X-ray nano-tomography images using the method as reported^40^. Fourier-transform infrared (FTIR) spectra were recorded by UATR Two FT-IR spectrometer. X-ray photoelectron spectroscopy (XPS) was measured by PHI Quantera II (Ulvac-Phi Incorporation) photoelectron spectrometer with Al Kα (1846.6 eV). Raman spectra measurements were carried out using a LabRAM HR Raman spectrometer (Horiba Jobin Yvon) with a 532 nm laser. Zeta potential was investigated by Zeta potential analyzer (Zetasizer Nano ZS90, Malvern, UK). Tensile and bending tests were conducted by using an Instron 5943 universal testing machine with a strain rate of 2 mm min^−1^ for stretching. The laser (HGTECH LSU3EA) used for cutting was focused with an objective lens with a focal length of 170 mm.

#### Datasets

The source domain data used in this work are from the steaming potential datasets built by experimental observations^51^. To refine the data coverage, we generated a series of fitting data as data augmentation based on the theoretical model^59–61^. The source domain streaming potential dataset contains a total of 81253 samples. The 2D-WEG generation performance dataset of the target domain contains a total of 3620 samples, which we collected experimentally by using a programmable constant temperature and humidity chamber.

#### Transfer learning framework

The TL framework was implemented using PyTorch^62^. The source model$:{{{{{{{{\boldsymbol{{{{{\mathcal{X}}}}}}}}}}}}}}\to {{{{{{{{\boldsymbol{{{{{\mathcal{R}}}}}}}}}}}}}}$ is a mapping from the parameter space ${{{{{{{{\boldsymbol{{{{{\mathcal{X}}}}}}}}}}}}}}$ of streaming potential to the generation performance space ${{{{{{{{\boldsymbol{{{{{\mathcal{R}}}}}}}}}}}}}}$, which consists of two components—Encoder and Decoder. We implemented the components of the transfer learning framework using fully connected layers. The Encoder$:{{{{{{{{\boldsymbol{{{{{\mathcal{X}}}}}}}}}}}}}}\to {{{{{{{{\boldsymbol{{{{{\mathcal{H}}}}}}}}}}}}}}$ maps the raw parameter of the streaming potential into a hidden vector $\vec{{{{{{{{\bf{h}}}}}}}}}\in {{{{{{{{\boldsymbol{{{{{\mathcal{H}}}}}}}}}}}}}}$, which is implemented by a 4-layer multilayer perceptron (MLP) with 16, 64, [64, 128] and [64, 128] neurons per layer, where [64, 128] represents the number of neurons in this layer is randomly selected in the range of 64–128. The Decoder$:{{{{{{{{\boldsymbol{{{{{\mathcal{H}}}}}}}}}}}}}}\to {{{{{{{{\boldsymbol{{{{{\mathcal{R}}}}}}}}}}}}}}$ is a mapping from knowledge representation $\vec{{{{{{{{\bf{h}}}}}}}}}$ in the latent space ${{{{{{{{\boldsymbol{{{{{\mathcal{H}}}}}}}}}}}}}}$ to the streaming potential performance $\vec{{{{{{{{\bf{r}}}}}}}}}\in {{{{{{{{\boldsymbol{{{{{\mathcal{R}}}}}}}}}}}}}}$, which is implemented by a 2-layer MLP, where the number of neurons per layer is [16, 64] and 2. All activation functions in the source model are ReLU. We use the source model to learn the relationship between the key parameters of the streaming potential on the output voltage and define the loss function as the mean square error (MSE) between the ground truth and predicted values of the normalized voltage. A total of 200 source models were pre-trained, and for each source model, 63% of the data from the dataset was randomly selected as the training set, and the remaining data were used as the valid set to verify the learned models during training. All parameters are initialized by using He initialization and we optimize the source model parameters over MSE loss with the Adam optimizer, using a mini-batch size of 16. The source model is trained for 500 epochs, with exponential learning rate decay from 10^−4^ to 10^−6^. To accommodate the volatility of the experimental data, a random Gaussian noise with a standard deviation of 0.005 is added to the steaming potential or streaming current data during training to make its distribution closer to the distribution of the 2D-WEG generation performance data.

The opt-model$:{{{{{{\boldsymbol{{{{{\mathcal{P}}}}}}}}}}}}\to {{{{{{\boldsymbol{{{{{\mathcal{G}}}}}}}}}}}}$ is a mapping from the parameter space ${{{{{{\boldsymbol{{{{{\mathcal{X}}}}}}}}}}}}$ of 2D-WEG to the generation performance (*V*_oc_ or *I*_sc_) space ${{{{{{{{\boldsymbol{{{{{\mathcal{R}}}}}}}}}}}}}}$, which has three components——NormLayer, Encoder, Decoder. The NormLayer$:{{{{{{{{\boldsymbol{{{{{\mathcal{P}}}}}}}}}}}}}}\to {{{{{{{{\boldsymbol{{{{{\mathcal{X}}}}}}}}}}}}}}$ is a learnable auto-normalization layer at the bottom of the opt-model to accommodate the order of magnitude mismatch between 2D-WEG generation performance data and streaming potential data, which multiplies each parameter individually by a learnable weight and plus a learnable bias, respectively, containing only 16 weights and 16 biases as parameters. The Encoder$:{{{{{{{{\boldsymbol{{{{{\mathcal{X}}}}}}}}}}}}}}\to {{{{{{{{\boldsymbol{{{{{\mathcal{H}}}}}}}}}}}}}}$ is a mapping from renormalized parameters space ${{{{{{{{\boldsymbol{{{{{\mathcal{X}}}}}}}}}}}}}}$ to the latent space ${{{{{{{{\boldsymbol{{{{{\mathcal{H}}}}}}}}}}}}}}$, which maintains the same architecture as the Encoder of the corresponding source model. The Decoder$:{{{{{{{{\boldsymbol{{{{{\mathcal{H}}}}}}}}}}}}}}\to {{{{{{{{\boldsymbol{{{{{\mathcal{G}}}}}}}}}}}}}}$ maps the hidden knowledge representation $\vec{{{{{{{{\bf{h}}}}}}}}}\in {{{{{{\boldsymbol{{{{{\mathcal{H}}}}}}}}}}}}$ to the *V*_oc_/*I*_sc_ $\in {{{{{{{{\boldsymbol{{{{{\mathcal{G}}}}}}}}}}}}}}$ of 2D-WEG, which is implemented by a 3-layer MLP and the number of neurons per layer is [32, 128], [16, 32] and 1, respectively. All activation functions in the opt-model are ReLU. Due to the similar physical mechanism background, the underlying features learned in the MLP layers at the bottom of the source model are reusable. We transfer the parameter weights and biases of the Encoder of the source model to the Encoder of opt-model as initialization parameters. The NormLayer and the Decoder are trained from scratch using experimental data, while the Encoder is only fine-tuned with the initial learning rate of each layer set to 10^−6^, 10^−6^, 3 × 10^−6^, and 5 × 10^−6^, respectively. Each opt-model is trained by randomly selecting 63% of the data from the 2D-WEG dataset as the training set and the remaining data are randomly divided into two sets, a validation set and a test set to evaluate the learned models during training and after convergence, respectively. During training, the learning rate of the NormLayer and the Decoder decays from 10^−4^ to 10^−6^, while the learning rate of the Encoder decays simultaneously.

The inputs to both the source model and opt-model are the eight characteristic parameters and their logarithmic transformations, i.e. $\vec{{{{{{\bf{x}}}}}}}=[T,\,{{{{{\rm{log }}}}}}T,{{{{{{\rm{RH}}}}}}},\,{{{{{\rm{log }}}}}}{{{{{{\rm{RH}}}}}}},{C},\,{{{{{\rm{log }}}}}}C,\tau,{{{{{\rm{log }}}}}}T,\zeta,\,{{{{{\rm{log }}}}}} | \zeta |,{l},\,{{{{{\rm{log }}}}}}l,{d},\, {{{{{\rm{log }}}}}}d,{P}+1,\, {{{{{\rm{log }}}}}}\left(P+1\right)]$, and the output is the streaming potential or the generation performance data. The inputs and outputs of both the source model and the opt-model are normalized using the sample mean and sample standard deviation of the respective data sets, which are pre-calculated using the entire datasets and stored in metadata.json.

In the candidate optimal parameter search phase of the iterative optimization strategy, the predictions of opt-models are used as the objective function and the differential evolution algorithm is used to explore candidate optimal parameter combinations. The differential evolution algorithm has a differential evolution strategy of DE/rand-to-best/1/bin, a binomial crossover rate of 0.7, a mutation constant (scaling factor) of 1, a population size of 10,000, and the maximum number of generations for population evolution is 100,000. In the 2D-WEG reconstruction phase of the iterative optimization strategy, the optimal parameter combinations predicted by opt-models are used to prepare 2D-WEGs, and we recollect the power generation performance of 2D-WEGs with different structural parameter combinations. In the opt-model refinement phase of the iterative optimization strategy, newly collected experimental data are added to the 2D-WEG generation performance dataset, and the opt-model is retrained using the above training parameters.

## Supplementary information

Supplementary Information

Description of Additional Supplementary Files

Supplementary Movie 1

Supplementary Movie 2

Supplementary Movie 3

## Data Availability

Source data are provided with this paper.

## Source data

Source Data

## Code availability

The open-source implementation of this work is available at Github [https://github.com/THUAML/Transfer_Learning_Enhanced_Water-Enabled_Electricity_Generation].

## References

[1] Liu XM (2020). Power generation from ambient humidity using protein nanowires. Nature.

[2] Wang HY (2020). Bilayer of polyelectrolyte films for spontaneous power generation in air up to an integrated 1,000 V output. Nat. Nanotechnol..

[3] Xue GB (2017). Water-evaporation-induced electricity with nanostructured carbon materials. Nat. Nanotechnol..

[4] Dhiman P (2011). Harvesting energy from water flow over graphene. Nano Lett..

[5] van der Heyden FHJ, Stein D, Dekker C (2005). Streaming currents in a single nanofluidic channel. Phys. Rev. Lett..

[6] Qin BC (2021). Power generation and thermoelectric cooling enabled by momentum and energy multiband alignments. Science.

[7] Ren W (2021). High-performance wearable thermoelectric generator with self-healing, recycling, and Lego-like reconfiguring capabilities. Sci. Adv..

[8] Feng JD (2016). Single-layer MoS_2_ nanopores as nanopower generators. Nature.

[9] Liu YC, Yeh LH, Zheng MJ, Wu KCW (2021). Highly selective and high-performance osmotic power generators in subnanochannel frameworks enabled by metal-organic frameworks. Sci. Adv..

[10] Guo W (2010). Energy harvesting with single‐ion‐selective nanopores: a concentration‐gradient‐driven nanofluidic power source. Adv. Funct. Mater..

[11] Zhang R (2015). A streaming potential/current‐based microfluidic direct current generator for self‐powered nanosystems. Adv. Mater..

[12] Ma QL (2020). Rational design of MOF‐based hybrid nanomaterials for directly harvesting electric energy from water evaporation. Adv. Mater..

[13] Zhou SY, Qiu Z, Strømme M, Chao X (2021). Solar-driven ionic power generation via a film of nanocellulose @ conductive metal-organic framework. Energy Environ. Sci..

[14] Zhou XB (2020). Harvesting electricity from water evaporation through microchannels of natural wood. ACS Appl. Mater. Interfaces.

[15] Fan B, Bhattacharya A, Bandaru PR (2018). Enhanced voltage generation through electrolyte flow on liquid-filled surfaces. Nat. Commun..

[16] Wang L, Wang ZX, Patel SK, Lin SH, Elimelech M (2021). Nanopore-based power generation from salinity gradient: why it is not viable. ACS Nano.

[17] Tong X, Liu S, Crittenden J, Chen YS (2021). Nanofluidic frameworks to address the challenges of salinity gradient power harvesting. ACS Nano.

[18] Su JJ (2018). Anomalous pore‐density dependence in nanofluidic osmotic power generation. Chin. J. Chem..

[19] Ghosh S, Sood AK, Ramaswamy S, Kumar N (2004). Flow-induced voltage and current generation in carbon nanotubes. Phys. Rev. B.

[20] Yoon SG (2021). Evaporative electrical energy generation via diffusion-driven ion-electron-coupled transport in semiconducting nanoporous channel. Nano Energy.

[21] Schoch RB, Han J, Renaud P (2008). Transport phenomena in nanofluidics. Rev. Mod. Phys..

[22] Daiguji H, Yang PD, Szeri AJ, Majumdar A (2004). Electrochemomechanical energy conversion in nanofluidic channels. Nano Lett..

[23] Dao VD, Vu NH, Choi HS (2020). All day Limnobium laevigatum inspired nanogenerator self-driven via water evaporation. J. Power Sources.

[24] Bae, J. et al. Towards Watt-scale hydroelectric energy harvesting by Ti_3_C_2_T_*x*_-based transpiration-driven electrokinetic power generators. *Energy Environ. Sci*. 10.1039/d1ee00859e (2021).

[25] van der Heyden FHJ, Bonthuis DJ, Stein D, Meyer C, Dekker C (2007). Power generation by pressure-driven transport of ions in nanofluidic channels. Nano Lett..

[26] van der Heyden FHJ, Bonthuis DJ, Stein D, Meyer C, Dekker C (2006). Electrokinetic energy conversion efficiency in nanofluidic channels. Nano Lett..

[27] Li LH (2020). Sustainable and flexible hydrovoltaic power generator for wearable sensing electronics. Nano Energy.

[28] Liu K (2018). Thermal–electric nanogenerator based on the electrokinetic effect in porous carbon film. Adv. Energy Mater..

[29] Ding TP (2017). All‐printed porous carbon film for electricity generation from evaporation‐driven water flow. Adv. Funct. Mater..

[30] Yang C, Huang YX, Cheng HH, Jiang L, Qu LT (2019). Rollable, stretchable, and reconfigurable graphene hygroelectric generators. Adv. Mater..

[31] Ma HY (2019). Highly ordered graphene solid: an efficient platform for capacitive sodium-ion storage with ultrahigh volumetric capacity and superior rate capability. ACS Nano.

[32] Zhang MC (2019). Controllable ion transport by surface-charged graphene oxide framework. Nat. Commun..

[33] Huang YX (2019). All-region-applicable, continuous power supply of graphene oxide composite. Energy Environ. Sci..

[34] Huang YX (2018). Interface-mediated hygroelectric generator with an output voltage approaching 1.5 volts. Nat. Commun..

[35] Cheng HH (2018). Spontaneous power source in ambient air of a well-directionally reduced graphene oxide bulk. Energy Environ. Sci..

[36] Liu ZY, Jiang M, Luo TF (2020). Leverage electron properties to predict phonon properties via transfer learning for semiconductors. Sci. Adv..

[37] Gupta V (2021). Cross-property deep transfer learning framework for enhanced predictive analytics on small materials data. Nat. Commun..

[38] Zhong J (2018). Efficient and scalable synthesis of highly aligned and compact two-dimensional nanosheet films with record performances. Nat. Commun..

[39] Zhang PP (2018). Three-dimensional water evaporation on a macroporous vertically aligned graphene pillar array under one sun. J. Mater. Chem. A.

[40] Chen-Wiegart YK (2014). Tortuosity characterization of 3D microstructure at nano-scale for energy storage and conversion materials. J. Power Sources.

[41] Lv ZS (2018). Editable supercapacitors with customizable stretchability based on mechanically strengthened ultralong MnO_2_ nanowire composite. Adv. Mater..

[42] Tabrizizadeh T (2021). Water-evaporation-induced electric generator built from carbonized electrospun polyacrylonitrile nanofiber mats. ACS Appl. Mater. Interfaces.

[43] Yun TG, Bae J, Rothschild A, Kim ID (2019). Transpiration driven electrokinetic power generator. ACS Nano.

[44] Qin YS (2020). Constant electricity generation in nanostructured silicon by evaporation‐driven water flow. Angew. Chem. Int. Ed..

[45] Das SS, Pedireddi VM, Bandopadhyay A, Saha P, Chakraborty S (2019). Electrical power generation from wet textile mediated by spontaneous nanoscale evaporation. Nano Lett..

[46] Wu M (2021). Printed honeycomb-structured reduced graphene oxide film for efficient and continuous evaporation-driven electricity generation from salt solution. ACS Appl. Mater. Interfaces.

[47] Lee KH, Kang DJ, Eom W, Lee H, Han TH (2022). Holey graphene oxide frameworks containing both nanopores and nanochannels for highly efficient harvesting of water evaporation energy. Chem. Eng. J..

[48] Ji BX (2019). Intelligent multiple-liquid evaporation power generation platform using distinctive Jaboticaba-like carbon nanosphere@TiO_2_ nanowires. J. Mater. Chem. A.

[49] Purja Pun GP, Batra R, Ramprasad R, Mishin Y (2019). Physically informed artificial neural networks for atomistic modeling of materials. Nat. Commun..

[50] Ripalda JM, Buencuerpo J, García I (2018). Solar cell designs by maximizing energy production based on machine learning clustering of spectral variations. Nat. Commun..

[51] Walker E, Glover PWJ (2018). Measurements of the relationship between microstructure, pH, and the streaming and Zeta potentials of sandstones. Transp. Porous Media.

[52] Tranter AD (2018). Multiparameter optimisation of a magneto-optical trap using deep learning. Nat. Commun..

[53] Knosgaard NR, Thygesen KS (2022). Representing individual electronic states for machine learning GW band structures of 2D materials. Nat. Commun..

[54] Sun JC (2019). Electricity generation from a Ni-Al layered double hydroxide-based flexible generator driven by natural water evaporation. Nano Energy.

[55] Wang ZY (2021). Hierarchical oriented metal-organic frameworks assemblies for water‐evaporation induced electricity generation. Adv. Funct. Mater..

[56] Guo Z (2022). Achieving steam and electrical power from solar energy by MoS_2_-based composites. Chem. Eng. J..

[57] Zhao YC (2008). Individual water‐filled single‐walled carbon nanotubes as hydroelectric power converters. Adv. Mater..

[58] Liu Z (2010). Surface‐energy generator of single‐walled carbon nanotubes and usage in a self‐powered system. Adv. Mater..

[59] Glover PWJ, Walker E, Jackson MD (2012). Streaming-potential coefficient of reservoir rock: a theoretical model. Geophysics.

[60] Glover PWJ (2018). Modelling pH-dependent and microstructure-dependent streaming potential coefficient and Zeta potential of porous sandstones. Transp. Porous Media.

[61] Thanh LD, Do PV, Nghia NV, Ca NX (2018). A fractal model for streaming potential coefficient in porous media. Geophys. Prospect..

[62] Paszke, A. et al. PyTorch: an imperative style, high-performance deep learning library. In *Proc. Advances in Neural Information Processing Systems*, (eds Wallach, H. et al.) Vol. 32 (Curran Associates, Inc., 2019).

