# Supplementary material for: Transfer learning enhanced water-enabled electricity generation in highly oriented graphene oxide nanochannels
Source: Nat Commun. 2022 Nov 10;13:6819. doi: 10.1038/s41467-022-34496-y (PMC9649687; doi:10.1038/s41467-022-34496-y)
Supplement: Supplementary file 1 — Supplementary Information [file 41467_2022_34496_MOESM1_ESM.pdf]

Supplementary Information

Transfer learning enhanced water-enabled electricity generation in highly oriented graphene oxide nanochannels

Ce Yang[1], Haiyan Wang[1], Jiaxin Bai[1], Tiancheng He[1], Huhu Cheng[1]*, Tianlei Guang[1], Houze Yao[1], Liangti Qu[1]*

[1] Key Laboratory of Organic Optoelectronics & Molecular Engineering, Ministry of Education, Department of Chemistry; State Key Laboratory of Tribology in Advanced Equipment (SKLT); Laboratory of Flexible Electronics Technology, Tsinghua University, Beijing 100084, P. R. China.

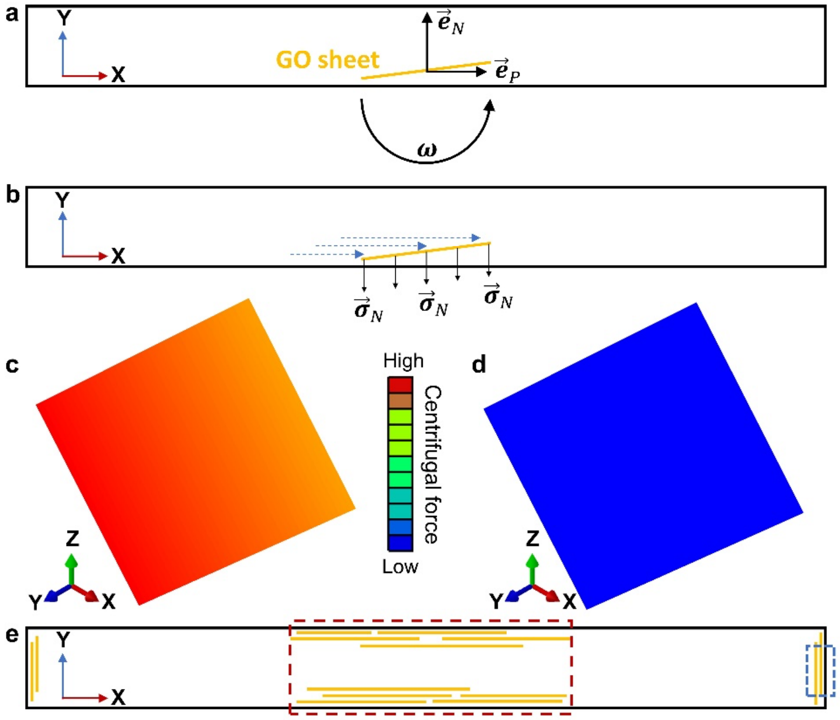

Supplementary Fig. 1. Force analysis of graphene oxide (GO) sheets during rotational freeze-casting process. (a) Schematic of a cross section of the rectangular mold for rotational freeze-casting method. (b) Analysis of the forces on the GO sheet during the rotational freeze-casting process. Distribution of the components of the centrifugal force on the GO sheet in the (c) **Y**-axis and (d) **X**-axis directions based on finite element analysis. (e) Schematic of the orientation distribution of GO sheets induced by rotational freeze-casting.

The cross section of the mold (Supplementary Fig. 1a) for the 2D GO assembled framework fabrication is about 20 cm long and 1.5 cm wide, and rotation process is around the central axis in the **Z**-axis direction. When the mold rotates, the GO sheet is subjected to radial centrifugal force $\vec{\sigma}_N$. The centrifugal force causes the GO sheets to tend to distribute approximately parallel to the mold walls due to the confinement of the mold walls[1]. In particular, for the GO sheet located in the central region of the long axis, the direction of the centrifugal force $\vec{\sigma}_N$ is essentially parallel to the tangential direction $\vec{e}_N$ of mold (Supplementary Fig. 1a and b), and the finite element analysis shows that the component of the centrifugal force in the **Y**-axis

direction (Supplementary Fig. 1c) is significantly larger than that in the **X**-axis direction (Supplementary Fig. 1d), which makes GO sheets tend to be distributed parallel to **X**-**Z** plane. Meanwhile, at the initial stage of rotation, the dispersion will form a flow parallel to the container wall, and the shear force of the flow will cause GO sheets to form an oriented distribution parallel to the flow direction[2,3]. As a result, GO sheets form an orientation distribution parallel to the mold wall, especially in the intermediate region of the long side and the short side (the red box and blue box indication area in Supplementary Fig. 1e). In this study, we choose GO assembled framework within 2 cm from the center of the long edge (the red box indication area in Supplementary Fig. 1e) to prepare 2D-WEG.

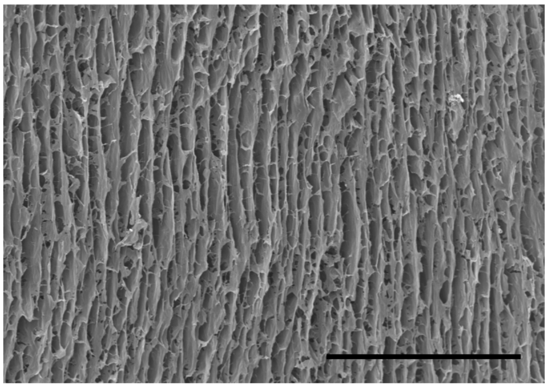

Supplementary Fig. 2. The cross-section scanning electron microscope (SEM) image of a GO assembled framework taken along the Z-axis direction. Scale bar: 500 μm.

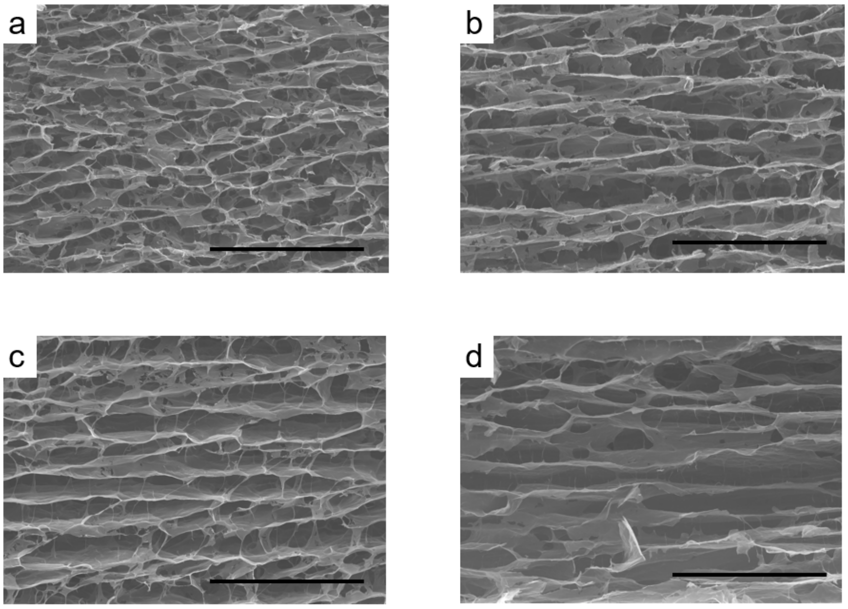

Supplementary Fig. 3. SEM images of cross-sections of GO assembled frameworks at different rotational speeds. SEM images of cross-sections in the **X-Y** plane of GO assembled frameworks prepared using rotational freeze-casting method at (a) 500, (b) 1000, (c) 1500, (d) 2000 rpm. Scale bars: 200 μm.

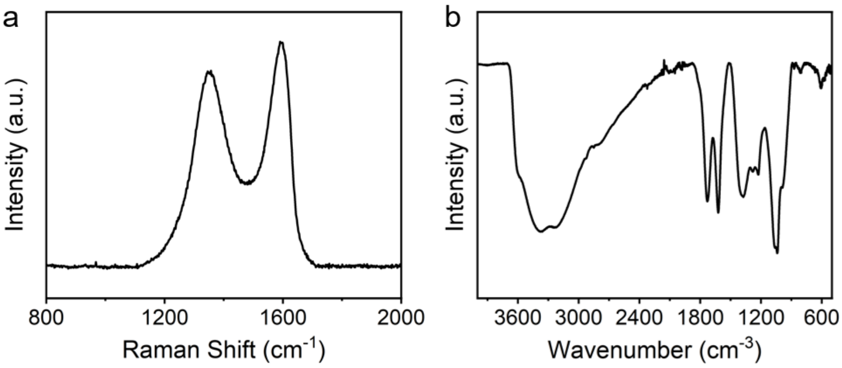

Supplementary Fig. 4. Chemical spectral analysis of GO assembled framework. (a) Raman spectra of the GO assembled framework indicates clear disorder D band (1350 cm^{-1}) and graphitic G band (1588 cm^{-1}) of GO. The calculated I_D/I_G ratio for the 2D-WEG is 0.88, which is consistent with reported studies[4,5]. (b) FT-IR spectrum of the GO assembled framework confirms the existence of abundant oxygen-containing functional groups. The broaden and intensive peak at 3380 cm^{-1} should be ascribed to -OH stretching. The sharp peak at 1731 cm^{-1} is caused by the stretching of C=O binding. The characteristic peak centered at 1226 cm^{-1} is associated with C–OH stretching. The obvious peak at 1045 cm^{-1} reveals stretching of C–O bonding[6,7].

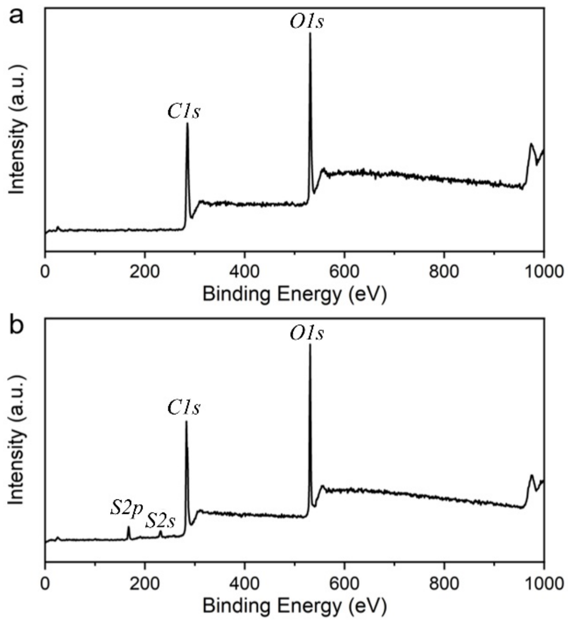

Supplementary Fig. 5. Chemical composition analysis of GO assembled frameworks. The XPS spectra of (a) GO assembled framework and (b) GO assembled framework doped with PSS indicate the chemical composition of 2D-WEG.

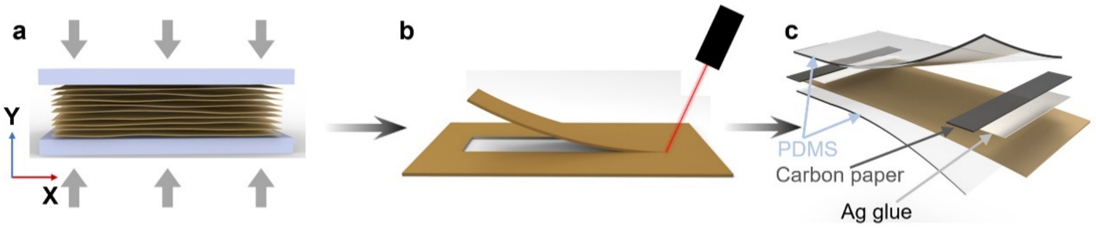

Supplementary Fig. 6. Fabrication process of the 2D-WEG. (a) Tableting process along the **Y**-axis to press GO assembled framework to a predetermined thickness. (b) Using a programmable laser to cut the GO assembled framework into designed shapes. (c) Using conductive silver glue to connect carbon paper electrodes to both ends of the GO assembled framework to collect electrical signals. Then, the two electrodes and the middle GO area were carefully sealed with a pre-formed PDMS film.

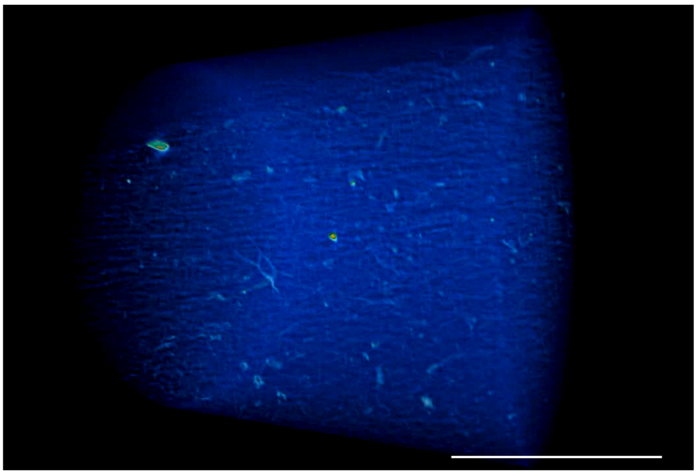

Supplementary Fig. 7. The 3D structure of the GO assembled framework in 2D-WEG after tableting processing is reconstructed based on X-ray tomography images. Scale bar: 30μm.

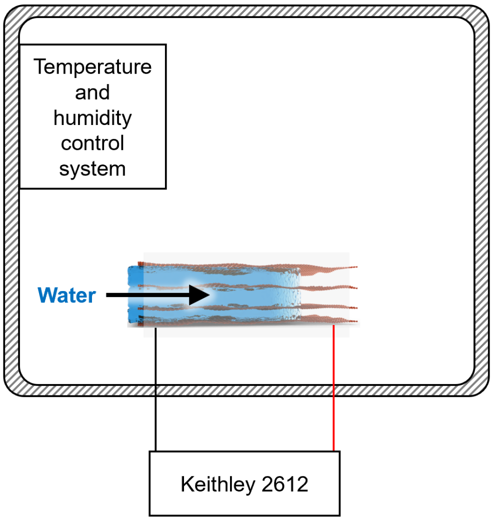

Supplementary Fig. 8. Schematic illustration of the temperature and humidity control system for electrical testing of 2D-WEGs. The 2D-WEG is placed in a constant temperature and humidity chamber to collect the electrical signals. If not specified, the temperature is controlled to fluctuate between 29 °C and 31 °C degrees and the relative humidity (RH) is controlled to fluctuate between 15% and 20%.

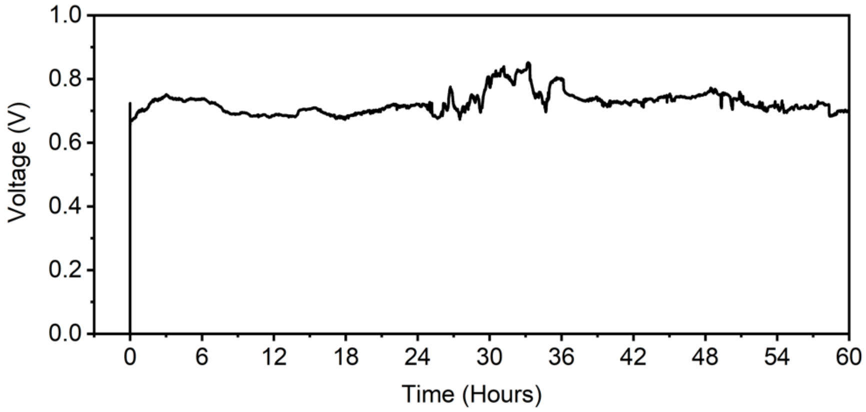

Supplementary Fig. 9. ***V_{oc}*** **generated by the as-prepared 2D-WEG is sustained for 60 hours.**

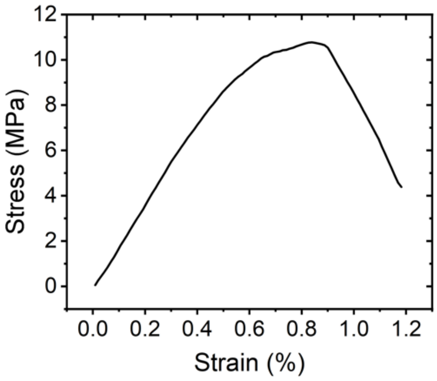

Supplementary Fig. 10. The stress-strain curve of the GO assembled framework.

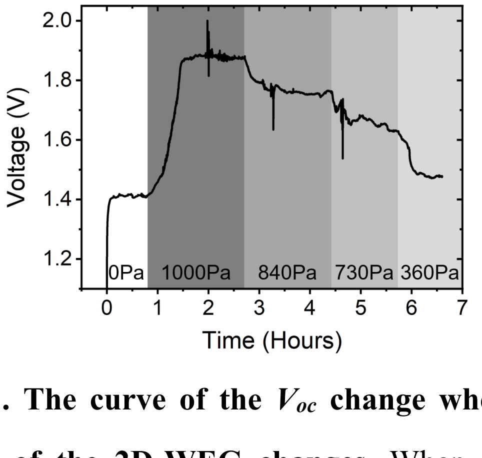

Supplementary Fig. 11. The curve of the ***V_{oc}*** **change when pressure difference between the two ends of the 2D-WEG changes.** When the applied pressure is increased, it accelerates the water flow through the 2D-WEG and the V_{oc}. When the pressure decreases, the V_{oc} also decreases due to the slowing down of the water flow.

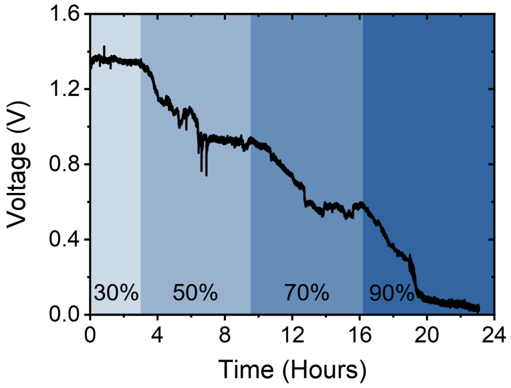

Supplementary Fig. 12. The curve of the V_{oc} change when RH changes. When the RH increases, the V_{oc} decreases sequentially, which shows the strong correlation between the energy conversion process and the water evaporation process.

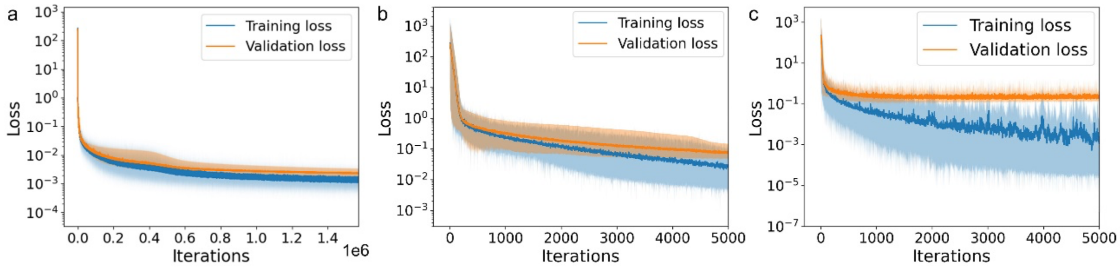

Supplementary Fig. 13. The loss function trace during training. The loss function trace of the training and validation sets during the training phase for (a) the source model as well as (b) opt-model with TL and (c) opt-model without TL. The curves show the average loss function traces over 20 independent trainings with the shaded area corresponding to the intervals of the maximum and minimum losses over 20 independent trainings in each iteration.

Supplementary Note 1

The controlled variable method can be adopted in the opt-model to analyze the influence of each parameter on 2D-WEG generation performance (Supplementary Table 1). As shown in Supplementary Fig. 14a, the numerical modeling results indicate both V_{oc} and I_{sc} of 2D-WEG can be enhanced as T increases (Supplementary Fig. 15). This should be attributed to that high temperature will promote the water transport and evaporation process and further facilitate energy conversion, which is

consistent with previous reports on other WEGs[8,9,10]. When *RH* reaches a high value of 90%, the V_{oc} and I_{sc} of 2D-WEG will decay to below 0.1 V and 0.1 μA, respectively (Supplementary Fig. 14b), which indicates a negative correlation between the power generation and *RH* that could affect the water evaporation behavior[8,10]. When additional *P* is applied to promote the water flow, the V_{oc} and I_{sc} can increase approximately linearly, providing evidence that the generation performance is correlated with the directional transmission of water (Supplementary Fig. 14c). Meanwhile, 2D-WEG with lower structure tortuosity τ should generate higher I_{sc} (Supplementary Fig. 14d), because the ordered texture can induce higher ion diffusion coefficient[11–14] (Supplementary Fig. 16). In addition, the resistance for water directional transport could also depend on the nanochannel spacing *d* from the numerical modeling results. Too small channel spacing *d* will hinder water directional transmission, resulting in the low V_{oc} and I_{sc} of 2D-WEG[13–17]. However, when the channel spacing *d* is too large, the low V_{oc} of 2D-WEG will be induced, supposedly because the formed EDL on nanochannels cannot be overlapped in the enlarged spacing as reported in literatures[8,10,18,19] (Supplementary Figs. 14e and 17). For the 2D nanochannels integration length *l*, the generated V_{oc} increases to be a highest value at the length of about 14.6 cm and then decreases with further increases of *l* (Supplementary Fig. 14f). This indicates the positive and negative charges separation induced electric potential difference in 2D-WEG could be enhanced at a certain distance under capillary force[8,20]. When *l* is longer than distance of capillary action for water absorption, water transport and evaporation will be gradually inhibited, resulting in the weakened V_{oc}[10,13,21]. Meanwhile, the overall resistance of 2D-WEG will increase when 2D nanochannels integration length increases, which would result in a decrease in I_{sc}[11]. Besides, as shown in Supplementary Fig. 14g, both V_{oc} and I_{sc} are enhanced as Zeta potential ζ increases, mainly because the ζ reflects the charge density of ions adsorbed in EDL, thus suggesting a best value of ζ (-83 mV) to pursue the maximum power output[10,13]. When ions concentration *C* increases, the V_{oc} and I_{sc} decrease sharply (Supplementary Fig. 14h), which should be because the increased ions concentration will weaken the EDL in 2D-WEG[10].

Supplementary Table 1. The values of each parameter in the control variables analysis in Supplementary Figs. 14.

	T (°C)	RH (%)	P (Pa)	τ
Supplementary Fig. 14a	30 ~ 90	15	0	1.04
Supplementary Fig. 14b	30	30 ~ 90	0	1.04
Supplementary Fig. 14c	30	15	360 ~ 1000	1.04
Supplementary Fig. 14d	30	15	0	1.04 ~ 4.37
Supplementary Fig. 14e	30	15	0	1.04
Supplementary Fig. 14f	30	15	0	1.04
Supplementary Fig. 14g	30	15	0	1.04
Supplementary Fig. 14h	30	15	0	1.04

	d (nm)	l (cm)	ζ (mV)	C (M)
Supplementary Fig. 14a	98	9	-83	0
Supplementary Fig. 14b	98	9	-83	0
Supplementary Fig. 14c	98	9	-83	0
Supplementary Fig. 14d	98	9	-83	0
Supplementary Fig. 14e	26 ~ 267	9	-83	0
Supplementary Fig. 14f	98	V_{oc} : 2.5 ~ 22<br>I_{sc} : 2.5 ~ 14.6	-83	0
Supplementary Fig. 14g	98	9	-31 ~ -83	0
Supplementary Fig. 14h	98	9	-83	0 ~ 4

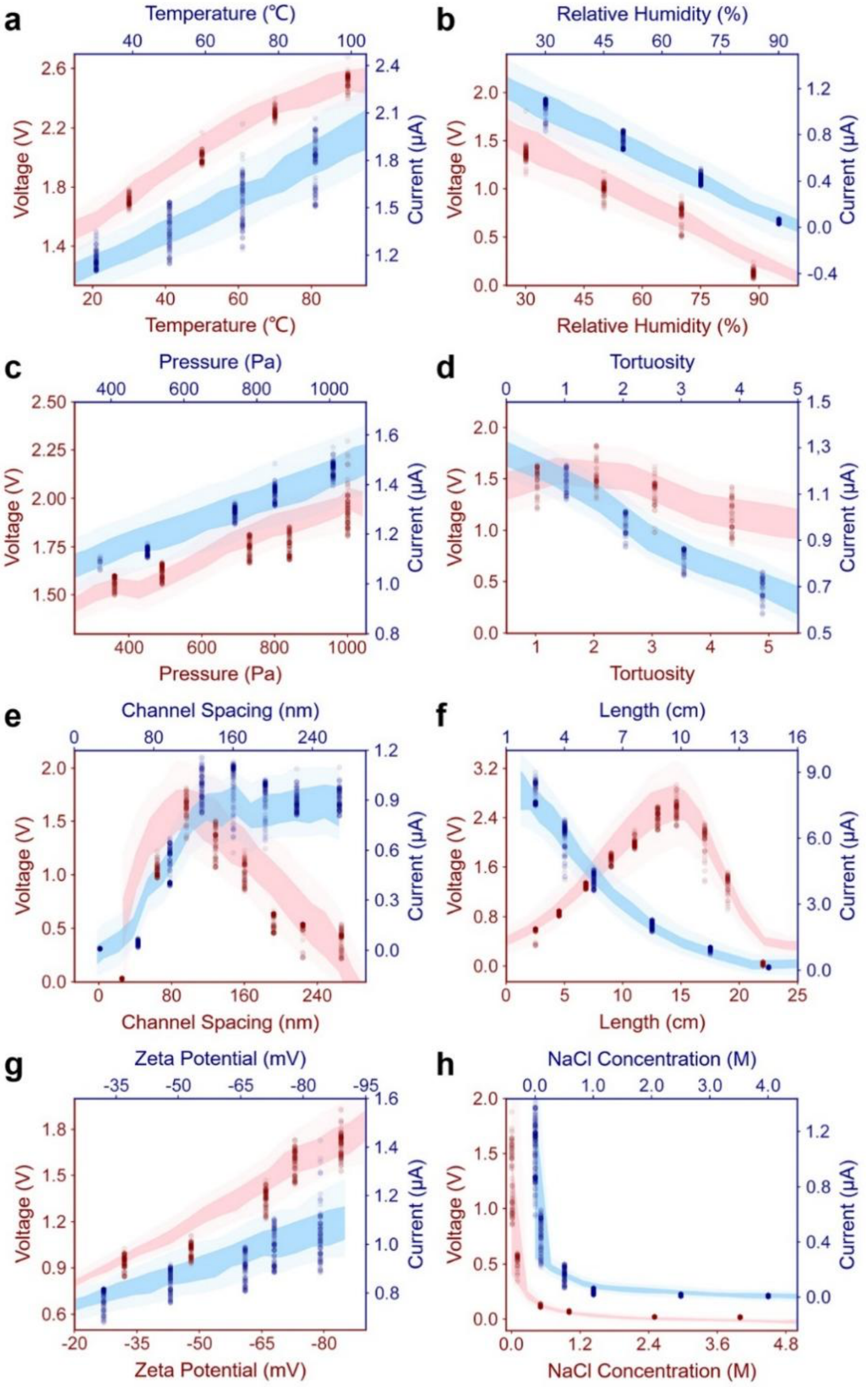

Supplementary Fig. 14. V_{oc}s and I_{sc}s at different characteristic parameters. The V_{oc} and I_{sc} of 2D-WEGs under different (a) liquid temperature T, (b) environmental relative humidity RH, (c) pressure driving P, (d) structural tortuosity τ, (e) channel spacing d, (f) device length l, (g) Zeta potential ζ and (h) NaCl concentration C. The translucent blue and red scatter dots indicate experimentally measured V_{oc} and I_{sc},

respectively. The two different transparency intervals represent the 50% and 95% confidence intervals of the opt-model predictions.

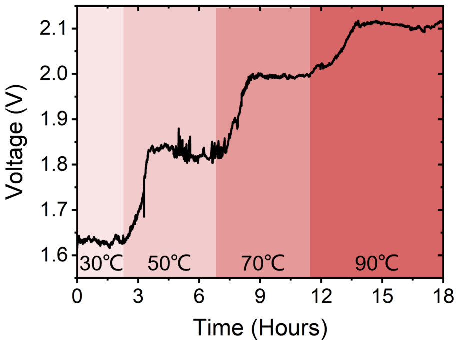

Supplementary Fig. 15. The curve of the V_{oc} change when water temperature changes.

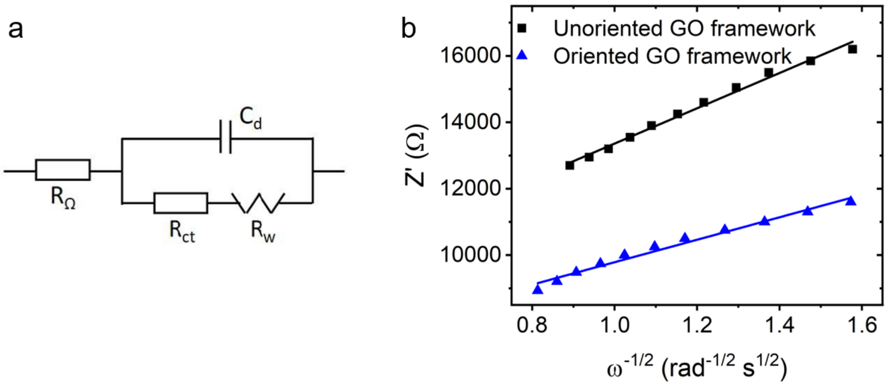

Supplementary Fig. 16. Analysis of ion diffusion coefficients of oriented and unoriented GO assembled frameworks. (a) Typical equivalent circuit for carbon-based materials. (b) Linear plots of real resistances (Z') against angular frequencies ($\omega^{-1/2}$) in the low frequency region.

The equivalent series resistance for carbon-based materials is mainly composed of three parts: intrinsic ohmic resistance (R_{Ω}), interfacial charge transfer resistance (R_{ct}), and Warburg diffusion resistance (R_W). Specifically, R_W represents the resistance to ion diffusion in the electrolyte and the ion diffusion coefficient can be calculated by using the following equation[22]:

$$D = \frac{R^2 T_K^2}{2n^4 F^4 A^2 C_m^2 \sigma^2}$$

where D is the ion diffusion coefficient, R is the gas constant, T_K is the absolute temperature, n is the electron number, A is the geometric area of electrode, F is the Faraday constant, and C_m is the molar concentration of ions. Additionally, σ is the Warburg coefficient, which can be calculated by plotting Z' against $\omega^{-1/2}$ on the basis of the equation of $Z' = R_{\Omega} + R_{ct} + \sigma\omega^{-1/2}$. As shown in Supplementary Fig. 16b, the calculated Warburg coefficient of the unoriented GO assembled framework is approximately 1.57 times higher than the coefficient of the oriented GO assembled framework. As a result, the ion diffusion coefficient of oriented GO assembled framework is about 2.46 times higher than that of the unoriented GO assembled framework.

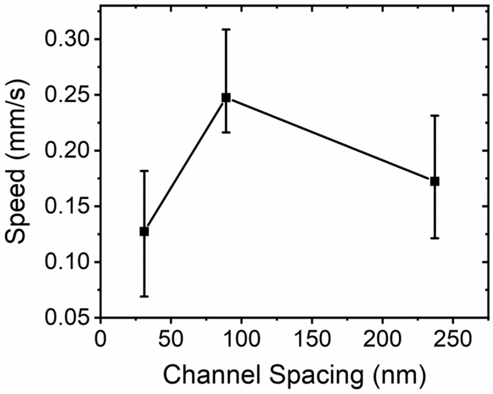

Supplementary Fig. 17. Water transport rate inside 2D-WEGs with different nanochannel spacing. The error bars represent deviations from multiple measurements.

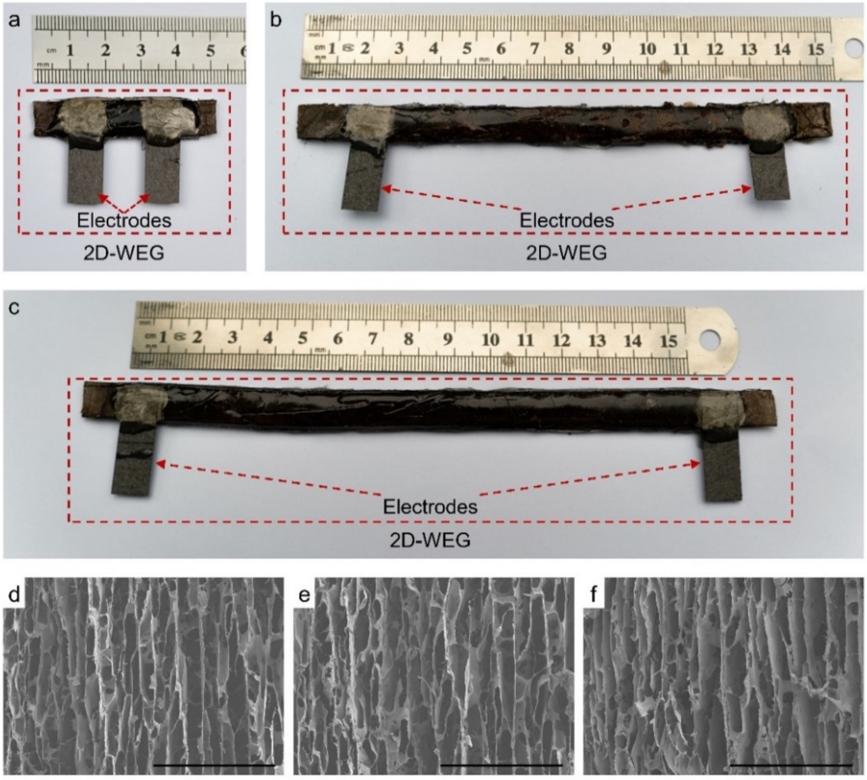

Supplementary Fig. 18. Optical photos and SEM images of cross-sections of 2D-WEGs with different lengths. Optical photos of 2D-WEGs with lengths of (a) 1 cm, (b) 10 cm and (c)14.6 cm. SEM images of cross sections of GO assembled frameworks employed for 2D-WEGs with lengths of (d) 1 cm, (e) 10 cm, (f) 14.6 cm. Scale bars: 300 μm.

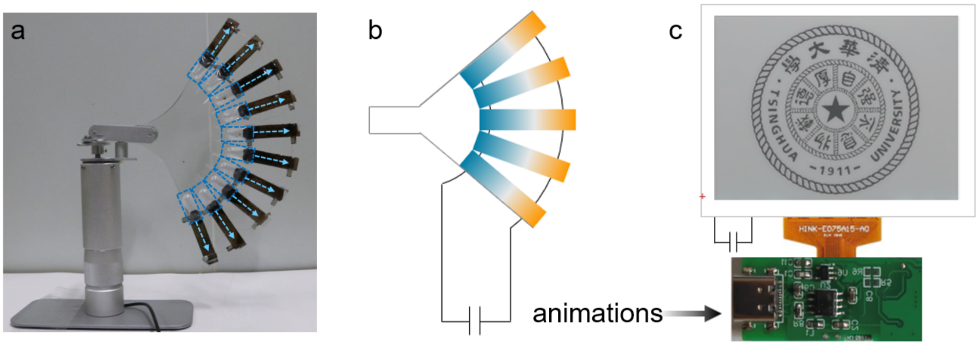

Supplementary Fig. 19. Optical photo of 2D-WEG fan and application demonstration schematic. (a) The optical photo of a mechanism device used to automatically swing a fan with ten integrated 2D-WEGs to promote the generated electricity. The blue boxes mark the water tanks used to supply water to 2D-WEGs and the blue arrows indicates the direction of water flow inside the 2D-WEGs. (b)

Schematic diagram of the designed circuit used to collect electricity from the waving of the fan and (c) the working demonstration of the self-powered electronic ink screen. The circuit primarily consists of 3 elements: an electronic ink screen, a capacitor for power supply and a single-chip computer to input the display message. The electrical energy that drives the animation on the electronic ink screen is generated by waving the fan with the integrated 2D-WEGs.

Supplementary Table 2. Summary of open - circuit voltage and short - circuit current of water-enabled electric generators. Here, we have removed water-enabled electric generators that use active metals.

Voltage (V)	Current (μA)	Reference
0.28	13	23
0.3	10	24
0.7	1.3	25
0.89	0.4	26
1	0.15	27
0.9	0.6	28
1.63	0.4	29
0.6	0.12	30
0.284	9.36	31
0.83	2.37	32
0.8	0.3	33
0.5	7	34
0.44	0.2	35
0.33	6.7	36
0.712	0.372	37
0.53	3.91	38
0.03	3	39
3	0.00012	40

0.16	0.0025	41
0.01	0.01	42
0.000382	1.42	43

Supplementary references

1. Zhong, J., *et al*. Efficient and scalable synthesis of highly aligned and compact two-dimensional nanosheet films with record performances. *Nat. Commun.* 9, 3484 (2018).
2. Xu, Z. & Gao, C. Graphene in Macroscopic Order: Liquid Crystals and Wet-Spun Fibers. *Acc. Chem. Res.* 47, 1267-1276 (2014).
3. Jalili, R., *et al*. Processable 2D materials beyond graphene: MoS_2 liquid crystals and fibres. *Nanoscale* 8, 16862-16867 (2016).
4. Yang, D., *et al*. Chemical analysis of graphene oxide films after heat and chemical treatments by X-ray photoelectron and Micro-Raman spectroscopy. *Carbon* **47**, 145-152 (2009).
5. Malard, L. M., Pimenta, M. A., Dresselhaus, G. & Dresselhaus, M. S. Raman spectroscopy in graphene. *Phys. Rep.* **473**, 51-87 (2009).
6. Xu, Y. X., Bai, H., Lu, G. W., Li, C. & Shi, G. Q. Flexible graphene films via the filtration of water-soluble noncovalent functionalized graphene sheets. *J. Am. Chem. Soc.* **130**, 5856-5857 (2008)
7. Dreyer, D. R., Park, S., Bielawski, C. W. & Ruoff, R. S. The chemistry of graphene oxide. *Chem. Soc. Rev.* **39**, 228-240 (2010).
8. Li, L. H., *et al*. Sustainable and flexible hydrovoltaic power generator for wearable sensing electronics. *Nano Energy*, **72**, 104663 (2020).
9. Liu, K., *et al*. Thermal–electric nanogenerator based on the electrokinetic effect in porous carbon film. *Adv. Energy Mater.* **8**, 1702481 (2018).
10. Qin Y. S., *et al*. Constant electricity generation in nanostructured silicon by evaporation-driven water flow. *Angew. Chem. Int. Ed.* **132**, 10706-10712 (2020).
11. Zhou, X. B., *et al*. Harvesting electricity from water evaporation through microchannels of natural wood. *ACS Appl. Mater. Interfaces* **12**, 11232-11239

(2020).

12. Zhou, S. Y., Qiu, Z., Strømme, M. & Chao, X. Solar-driven ionic power generation via a film of nanocellulose @ conductive metal–organic framework. *Energy Environ. Sci.* **14**, 900-905 (2021).
13. Zhang, Z. H., *et al*. Emerging hydrovoltaic technology. *Nat. Nanotechnol.* **13**, 1109-1119 (2018).
14. Dai, H. W., Xu, Z. J. & Yang, X. N. Water permeation and ion rejection in layer-by-layer stacked graphene oxide nanochannels: a molecular dynamics simulation. *J. Phys. Chem. C* **120**, 22585-22596 (2016).
15. van der Heyden, F. H. J., Stein, D. & Dekker, C. Streaming currents in a single nanofluidic channel. *Phys. Rev. Lett.* **95**, 116104 (2005).
16. van der Heyden, F. H. J., Bonthuis, D. J., Stein, D., Meyer, C. & Dekker, C. Power generation by pressure-driven transport of ions in nanofluidic channels. *Nano Lett.* **7**, 1022-1025 (2007).
17. Lee, K. H., Kang, D. J., Eom, W., Lee, H. & Han, T. H. Holey graphene oxide frameworks containing both nanopores and nanochannels for highly efficient harvesting of water evaporation energy. *Chem. Eng. J.* **430**, 132759 (2022).
18. Fan, B., Bhattacharya, A. & Bandaru. P. R. Enhanced voltage generation through electrolyte flow on liquid-filled surfaces. *Nat. Commun.* **9**, 4050 (2018).
19. Daiguji, H., Yang, P. D., Szeri, A. J. & Majumdar, A. Electrochemomechanical energy conversion in nanofluidic channels. *Nano Lett.* **4**, 2315-2321 (2004).
20. Xue, G. B., *et al*. Water-evaporation-induced electricity with nanostructured carbon materials. *Nat. Nanotechnol.* **12**, 317-321 (2017).
21. Tabrizizadeh, T., *et al*. Water-evaporation-induced electric generator built from carbonized electrospun polyacrylonitrile nanofiber mats. *ACS Appl. Mater. Interfaces* **13**, 50900-50910 (2021).
22. Ma, H. Y., *et al*. Highly ordered graphene solid: an efficient platform for capacitive sodium-ion storage with ultrahigh volumetric capacity and superior rate capability. *ACS Nano* **13**, 9161-9170 (2019).
23. Qin Y. S., *et al*. Constant electricity generation in nanostructured silicon by

evaporation-driven water flow. *Angew. Chem. Int. Ed.* **132**, 10706-10712 (2020).

24. Zhou, X. B., *et al.* Harvesting electricity from water evaporation through microchannels of natural wood. *ACS Appl. Mater. Interfaces* **12**, 11232-11239 (2020).
25. Sun, J. C., *et al.* Electricity generation from a Ni-Al layered double hydroxide-based flexible generator driven by natural water evaporation. *Nano Energy* **57**, 269-278 (2019).
26. Liu, K., *et al.* Thermal–electric nanogenerator based on the electrokinetic effect in porous carbon film. *Adv. Energy Mater.* **8**, 1702481 (2018).
27. Xue, G. B., *et al.* Water-evaporation-induced electricity with nanostructured carbon materials. *Nat. Nanotechnol.* **12**, 317-321 (2017).
28. Ding, T. P., *et al.* All-printed porous carbon film for electricity generation from evaporation-driven water flow. *Adv. Funct. Mater.* **27**, 1700551 (2017).
29. Ma, Q. L., *et al.* Rational design of MOF-based hybrid nanomaterials for directly harvesting electric energy from water evaporation. *Adv. Mater.* **32**, 2003720 (2020).
30. Wang, Z. Y., *et al.* Hierarchical oriented metal–organic frameworks assemblies for water-evaporation induced electricity generation. *Adv. Funct. Mater.* **31**, 2104732 (2021).
31. Tabrizizadeh, T., *et al.* Water-evaporation-induced electric generator built from carbonized electrospun polyacrylonitrile nanofiber mats. *ACS Appl. Mater. Interfaces* **13**, 50900-50910 (2021).
32. Wu, M., *et al.* Printed honeycomb-structured reduced graphene oxide film for efficient and continuous evaporation-driven electricity generation from salt solution. *ACS Appl. Mater. Interfaces* **13**, 26989-26997 (2021).
33. Yoon, S. G, *et al.* Evaporative electrical energy generation via diffusion-driven ion-electron-coupled transport in semiconducting nanoporous channel. *Nano Energy* **80**, 105522 (2021).
34. Zhou, S. Y., Qiu, Z., Strømme, M. & Chao, X. Solar-driven ionic power generation via a film of nanocellulose @ conductive metal–organic framework. *Energy*

Environ. Sci. **14**, 900-905 (2021).

35. Lee, K. H., Kang, D. J., Eom, W., Lee, H. & Han, T. H. Holey graphene oxide membranes containing both nanopores and nanochannels for highly efficient harvesting of water evaporation energy. *Chem. Eng. J.* **430**, 132759 (2022).
36. Guo, Z, Z., *et al.* Achieving steam and electrical power from solar energy by MoS_2-based composites. *Chem. Eng. J.* **427**, 131008 (2022).
37. Das, S. S., Pedireddi, V. M., Bandopadhyay, A., Saha, P. & Chakraborty, S. Electrical power generation from wet textile mediated by spontaneous nanoscale evaporation. *Nano Lett.* **19**, 7191-7200 (2019).
38. Yun, T. G., Bae, J., Rothschild, A. & Kim, I. D. Transpiration driven electrokinetic power generator. *ACS Nano* **13**, 12703-12709 (2019).
39. Dhiman, P., *et al.* Harvesting energy from water flow over graphene. *Nano Lett.* **11**, 3123-3127 (2011).
40. van der Heyden, F. H. J., Bonthuis, D. J., Stein, D., Meyer, C. & Dekker, C. Power generation by pressure-driven transport of ions in nanofluidic channels. *Nano Lett.* **7**, 1022-1025 (2007).
41. Zhang R., *et al.* A streaming potential/current-based microfluidic direct current generator for self-powered nanosystems. *Adv. Mater.* **27**, 6482-6487 (2015).
42. Zhao, Y. C., *et al.* Individual water-filled single-walled carbon nanotubes as hydroelectric power converters. *Adv. Mater.* **20**, 1772-1776 (2008).
43. Liu, Z., *et al.* Surface-energy generator of single-walled carbon nanotubes and usage in a self-powered system. *Adv. Mater.* **22**, 999-1003 (2010).
