# Supplementary material for: Transfer learning enhanced water-enabled electricity generation in highly oriented graphene oxide nanochannels
Source: Nat Commun. 2022 Nov 10;13:6819. doi: 10.1038/s41467-022-34496-y (PMC9649687; doi:10.1038/s41467-022-34496-y)
Supplement: Supplementary file 2 — Description of Additional Supplementary Files [file 41467_2022_34496_MOESM2_ESM.pdf]

Description of Additional Supplementary Files

File Name: Supplementary Movie 1
Description: LED array powered by waterscape screen with integrated 2D-WEGs.

File Name: Supplementary Movie 2
Description: Commercial calculator powered by a 2D-WEG.

File Name: Supplementary Movie 3
Description: Dynamic electronic ink screen powered by integrated 2D-WEGs.
